# *acal* is a Long Non-coding RNA in JNK Signaling in Epithelial Shape Changes during Drosophila Dorsal Closure

**DOI:** 10.1371/journal.pgen.1004927

**Published:** 2015-02-24

**Authors:** Luis Daniel Ríos-Barrera, Irene Gutiérrez-Pérez, María Domínguez, Juan Rafael Riesgo-Escovar

**Affiliations:** 1 Instituto de Neurobiología, Universidad Nacional Autónoma de México, campus UNAM Juriquilla, Querétaro, México; 2 Instituto de Neurociencias, Universidad Miguel Hernández-CSIC, Campus de San Juan, Sant Joan d’Alacant, Alicante, España; Harvard Medical School, Howard Hughes Medical Institute, UNITED STATES

## Abstract

Dorsal closure is an epithelial remodeling process taking place during Drosophila embryogenesis. JNK signaling coordinates dorsal closure. We identify and characterize *acal* as a novel negative dorsal closure regulator. *acal* represents a new level of JNK regulation. The *acal* locus codes for a conserved, long, non-coding, nuclear RNA. Long non-coding RNAs are an abundant and diverse class of gene regulators. Mutations in *acal* are lethal. *acal* mRNA expression is dynamic and is processed into a collection of 50 to 120 bp fragments. We show that *acal* lies downstream of raw, a pioneer protein, helping explain part of raw functions, and interacts genetically with *Polycomb. acal* functions in trans regulating mRNA expression of two genes involved in JNK signaling and dorsal closure: *Connector of kinase to AP1 (Cka)* and *anterior open (aop). Cka* is a conserved scaffold protein that brings together JNK and Jun, and *aop* is a transcription factor. Misregulation of *Cka* and *aop* can account for dorsal closure phenotypes in *acal* mutants.

## Author Summary

Changes in cell shape affect many critical cellular and bodily processes, like wound healing and developmental events, and when gone awry, metastatic processes in cancer. Evolutionarily conserved signaling pathways govern regulation of these cellular changes. The Jun-N-terminal kinase pathway regulates cell stretching during wound healing and normal development. An extensively studied developmental process is embryonic dorsal closure in fruit flies, a well-established model for the regulation and manner of this cell shape changes. Here we describe and characterize a processed, long non-coding RNA locus, *acal*, that adds a new layer of complexity to the Jun-N-terminal kinase signaling, acting as a negative regulator of the pathway. *acal* modulates the expression of two key genes in the pathway: the scaffold protein Cka, and the transcription factor Aop. Together, they enable the proper level of Jun-N-terminal kinase pathway activation to occur to allow cell stretching and closure.

## Introduction

A large fraction of the eukaryotic genome codes for non-coding RNAs (ncRNAs), which are very abundant and diverse, yet mostly uncharacterized and of unknown functions [1,2]. Nevertheless, some are critical players in gene expression regulation. Non-coding RNAs encompass different classes of molecules. The best-characterized ncRNAs are small RNAs. Broadly, small RNAs recruit silencing machinery to mRNAs to inhibit translation and/or target them for degradation [3]. Small ncRNAs characterization was facilitated because they base-pair with cognate mRNA targets, and interact with common protein complexes to regulate gene expression [4].

In contrast, long non coding RNAs (lncRNAs) encode a diverse group of ncRNAs, which may be part of different molecular complexes, and can stimulate or inhibit gene expression [5]. Recognized lncRNAs range from 200 nucleotides to several kilobases [6]. Since they do not have evident sequence motifs for annotation, their identification and relevance has remained elusive. In addition, many have low expression levels, hindering isolation and characterization. Next-generation RNA sequencing uncovered many lncRNAs (over 50% of transcribed species [2,7]), increasing dramatically their number and repertoire [8]. Despite this, in most cases contribution of lncRNAs to gene expression regulation or other still awaits genetic and functional validation.

In *Drosophila melanogaster* some lncRNAs have been annotated [9], and play important roles in gene expression, as in vertebrates [10]. In spite of extensive genetic screens, forward genetics identification of lncRNAs has been very limited, as they are found to fine-tune gene expression with mild phenotypical contributions.

Here we characterize a Drosophila lncRNA with strong embryonic phenotypes and lethality. Mutations in this locus, *acal*, result in partially penetrant dorsal closure (DC) defects due to Jun N-terminal kinase (JNK) signaling over-activation. This results in failure of DC, leading to a lethal dorsal open phenotype.

During DC, lateral epidermal sheets stretch over a dorsal extra-embryonic cell layer, the amnioserosa, and fuse at the dorsal midline [11]. The JNK signaling pathway regulates DC, and is activated at the dorsalmost row of epidermal cells, the leading edge cells (LE), which act as a signaling center for DC ([12], reviewed in [13]).

In these epidermal cells, JNK activation induces expression of cytoskeleton and adhesion regulators for cell stretching [14]. JNK activation at the LE spreads the morphogenetic rearrangement by inducing the signaling ligand *decapentaplegic* (*dpp*), a fly homolog of vertebrate BMPs. Dpp signals to lateral epidermal cells and amnioserosa to promote cell shape changes [15,16]. This response induces stretching of lateral epithelia in absence of cell proliferation, and final zippering.

It is not known how JNK activation is triggered at the LE, nor completely known how LE restriction occurs. The JNK signaling pathway is an evolutionarily conserved, MAPK-type signaling pathway. It consists, at its core, of a cascade of kinases, from JN4K (the gene *misshapen* in flies), to JN3K *(slipper)*, JN2K *(hemipterous)*, and finally JNK proper *(basket)*. Activated JNK–bound to a scaffold protein called Connector of kinase to AP1 (Cka)–, phosphorylates the transcription factor Jra (Drosophila Jun). Jra, together with Kayak (Drosophila Fos), constitute the AP-1 transcription complex activating JNK target genes. Besides Jra, JNK also phosphorylates Anterior open (Aop), leading to Aop nuclear export, de-repressing JNK target genes. The JNK pathway is required for orchestration of embryonic dorsal closure, but also for wound healing and in response to certain stressful conditions [13].

During DC JNK activity is restrained from the lateral epidermis partly by *aop*, although this transcription factor also functions earlier in the tissue in a positive way by promoting epidermis differentiation, preventing ectopic mitoses [15]. The ‘*raw*-group genes’: *raw*, a pioneer protein, and *ribbon*, a transcription factor *(rib*, [17,18]) also restrict JNK activation in the lateral epithelia. Another member, *puckered (puc)*, coding for a JNK phosphatase, acts in a feedback loop in the LE, dephosphorylating active JNK, and stopping signaling. JNK activation is also antagonized in the amnioserosa by *pebbled (peb)*, which codes for a transcription factor [19].

We show here that *acal* lncRNA partly mediates Raw JNK signaling antagonism in the lateral epidermis. *acal* partakes in regulating expression of the scaffold protein Cka [20]. This explains partly *raw* function (*raw* as an *acal* regulator), and provides a framework for JNK activity down-regulation at the lateral epidermis during DC. We find that mutations in *acal* also alter the expression of *aop*, balancing JNK activation. *acal* also shows a genetic interaction with *Polycomb*, suggesting a relationship between *acal* and the Polycomb repressive complex. Overall, this provides a rationale for *acal* DC phenotypes.

## Results

### *acal* is a novel ‘dorsal open’ gene

**Figure 1:**
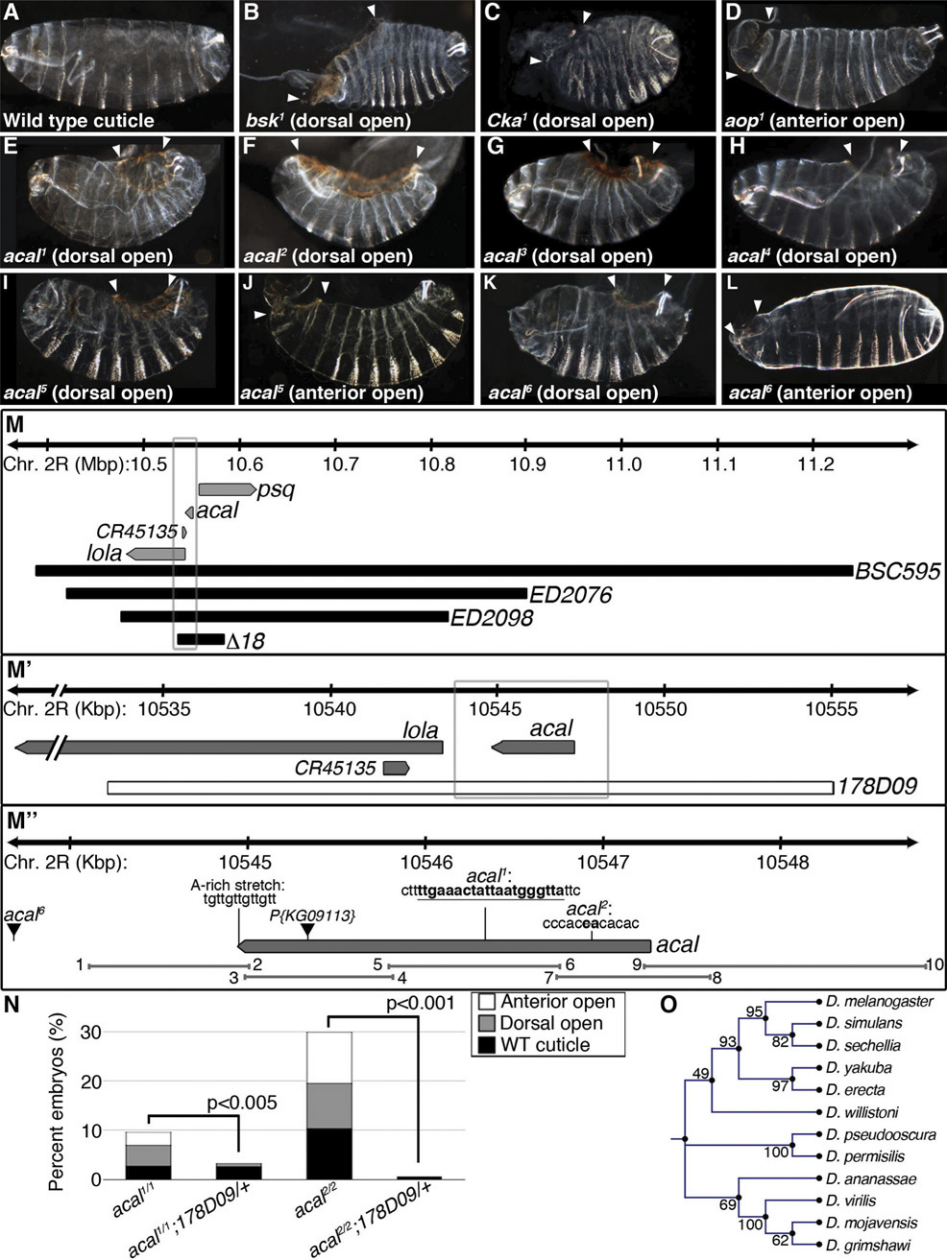
Molecular and genetic characterization of *acal* mutants. (A-L) Cuticular analysis of DC defects. In all figures, embryos are shown in a lateral view, with dorsal up and anterior left. Arrowheads show extent of cuticular holes. (A) Wild type cuticle. (B-D) *bsk^1^* (JNK), *Cka^1^*, and *aop^1^* mutant phenotypes. (E-L) *acal* mutants. (M-M’’) Schematic representation of the *acal* locus. (M) Deficiencies used to map *acal* mutants to the intergenic region between *lola* and *psq* (for simplicity, other genes are not depicted). Boxed area in (M) is amplified in (M’). (M’) The genomic rescue construct,*178D09*, spans the full *acal* genomic region, *CR45135*, and a small part of *lola* 5’. Boxed area in (M’) is amplified in (M’’). (M’’) Parental insertion *P{KG09113}* for *acal^1^* and *acal^2^* and insertion site of *acal^6^* are shown. Molecular lesions of *acal^1^* and *acal^2^* are highlighted in bold. A putative poly-adenylation site is also depicted. Primer pairs numbers and amplicons used to sequence mutant alleles are also depicted. (N) A genomic rescue transgene significantly suppresses *acal* DC mutant phenotypes. Only mutant embryos with cuticle defects are shown, mutants surviving embryogenesis are not depicted (and constitute the open space above bars to amount to a hundred percent). In this and all figures, unless noted, mutant embryos were selected by lack of GFP expression, present in control embryos (possessing balancer chromosomes that express GFP). Number of animals analyzed: *acal^1/1^* = 217, *acal^1/1^;178D09* = 314, *acal^2/2^* = 192, *acal^2/2^;178D09* = 159. Chi square tests were used to assess significance. (O) Similarity tree of *acal* homologs among some sequenced Drosophilids. Bootstrap values are shown. Further genetic characterization is shown in S1 Fig.

Mutations in fly JNK signaling genes—like *bsk*, the fly JNK gene [12], *Cka* [20], and *aop* [15]—lead to an embryonic lethal condition with a dorsal hole in the cuticle (Fig. 1B–D). This ‘dorsal open’ phenotype has been used to identify JNK and other DC genes [13].

We identified a lethal locus where a fraction of mutant embryos die with dorsal closure defects. We called it *acal*, or ‘boat’ in Náhuatl, due to the cuticular appearance of mutants. Two alleles, *acal^1^* and *acal^2^*(Fig. 1E–F), are lethal excisions of *P{KG09113}*, a viable and fertile P-element transposon insertion at 47A13 (Fig. 1M’’). Three more EMS alleles were isolated over *acal^1^* and *acal^2^* by lack of complementation (*acal^3^*, *acal^4^*, and *acal^5^*, Fig. 1G–J). A sixth lethal mutant allele is a mapped P element insertion in the locus, *P{GS325ND3}* (Fig. 1K-L and M’’). All mutations are lethal, with embryonic, larval, and pupal phenocritical periods. No adults are ever observed. A fraction of mutant embryos have DC defects, with holes in either the dorsal or anterior ends of embryos (Figs. 1E–L and S1A). Dorsal or anterior holes are the only cuticular defects seen in these mutants.

*acal* mutants have an extended phenocritical period, since a fraction of mutant embryos die without cuticular defects, and finally another fraction of mutant embryos survive embryogenesis and die later during larval stages up to the beginning of pupariation. This means that *acal* is required at various times during development. Larval lethality varies from 10 to 50% depending on the allele studied, and pupal death from 3 to 40%. As stated, no adult homozygous mutant flies are ever recovered, except 1–2% adult *acal^6^* escapers. In this work, we focus on the embryonic mutant phenotypes. Using these embryonic phenotypes, mutant alleles conform to an allelic series with *acal^5^* as the strongest, and *acal^1^* as the weakest (S1A Fig.).

### *acal* maps to an intergenic unannotated region and to SD08925

We used deficiencies uncovering *P{KG09113}* to map *acal* in complementation tests, and established a 100 kb interval where *acal* maps. This interval includes three annotated genes: *longitudinals lacking* (*lola*), *pipsqueak* (*psq*), and *CR45135* (Fig. 1M). *acal* mutations complement *lola* and *psq* alleles (S1 Table). Besides, *lola* and *psq* have different embryonic mutant phenotypes from *acal*, with no cuticular phenotypes, like dorsal or anterior holes ([21,22] and S7A Fig.). *CR45135* is not characterized, but partially overlaps a *lola* exon, so *CR45135* mutations should affect *lola*. Moreover, many *lola* lethal insertions overlap this gene [23].

A previous report showed that there is at least another locus whose mutation complements *lola* and *psq*, despite being unannotated [24]. This mutation, *l(2)00297*, suppresses the *peb^1^* heat-shock sensitive rough eye phenotype. *acal* mutant alleles also suppress, in our experimental conditions, this subtle but clear phenotype (S1B Fig.). This suggests that *acal* and *l(2)00297* are allelic, and is independent evidence of a lethal locus between *lola* and *psq*.

Another paper independently reports the existence of a long non-coding transcript approximately 4 kb from the start of *lola* [21]. The size of this transcript is stated as 4 kb, but this was an approximation, as the evidence supports a maximum size of 3.4 Kb, since this cDNA hybridized to a 2.8 and a 0.6 Kb neighboring restriction fragments in Southern blots ([21], and Edward Giniger, personal communication).

An EST in Flybase, SD08925, sequenced partially at the 5’ end, maps 3.8 kb away from *lola*, precisely where Giniger et al. report the location of their ‘alpha’ long non-coding transcript [21]. We believe this transcript is the same as SD08925. We sequenced fully this EST (Genbank accession number # KJ598082). SD08925 is 2.3 kb long, poly-adenylated, and a single exon. Its genomic 3’ end bears an A-rich stretch (Fig. 1M’’), characteristic of poly-adenylation sites [25]. We also obtained a 2.3 kb weak Northern blot signal (compared to control *Rp49)*, demonstrating SD08925 expression, and confirming that SD08925 is a full-length cDNA. This band was detected throughout the life cycle and was nearly absent in the *∆18* deficiency strain uncovering the locus by semi-quantitative RT-PCR (S1C-D Fig.). Consistent with the weak Northern signal, RNA-seq and microarray data show low SD08925 expression levels [26,27].

**Figure 2:**
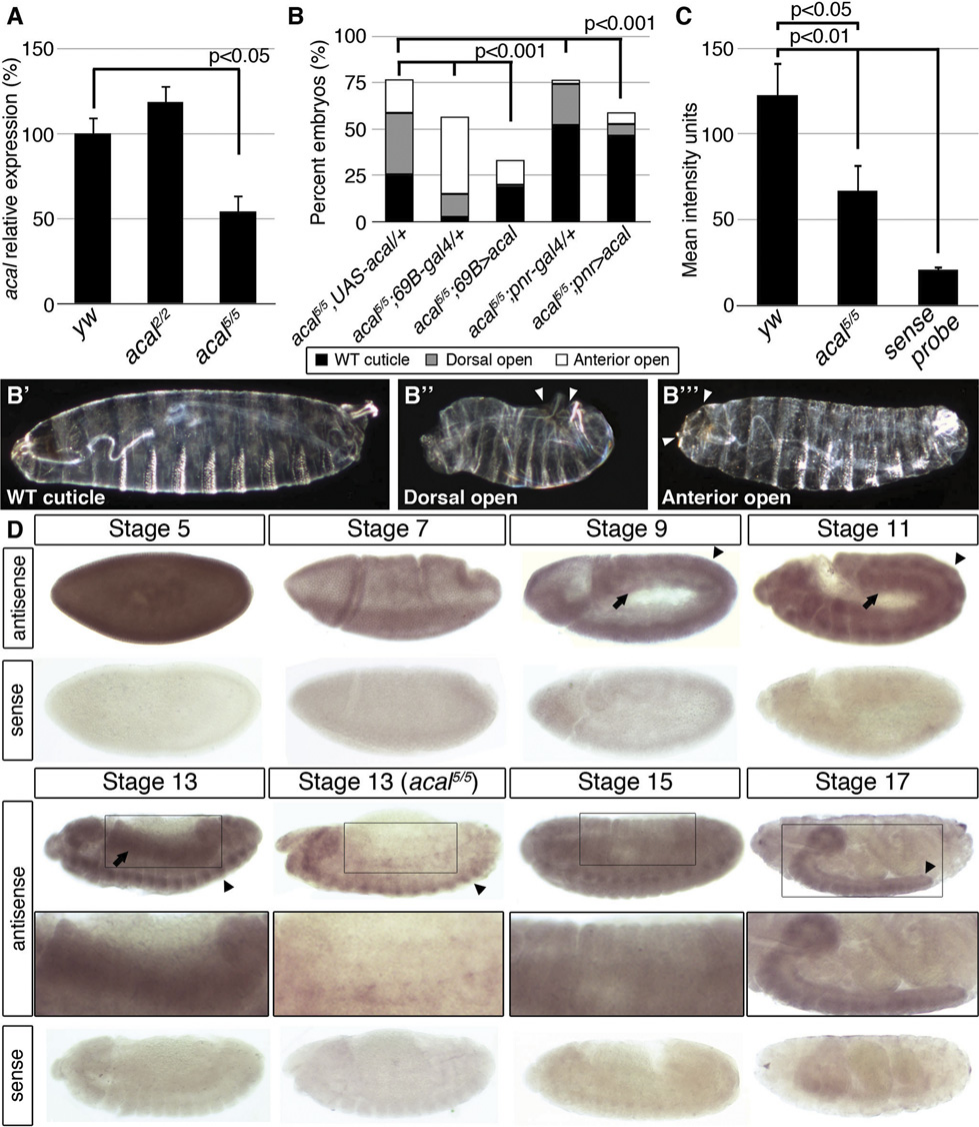
*acal* expression is required in the lateral epidermis during DC. (A) *acal* expression in wild type and mutant embryos during DC stages, as determined by qPCR. Means of three independent experiments run twice, +/− SEM. Student’s t test was used to assess significance. (B) Targeted ectodermal (*69B-gal 4* driver) and lateral epidermis (*pnr-gal 4* driver) expression of wild type *acal* in *acal* mutants. Only dead embryos are classified, mutants surviving embryogenesis constitute the remaining percentage to amount to a hundred percent (open space above bars). (B’-B’’’) are examples of *acal^5/5^; 69B>acal* embryos with no cuticular phenotype, wild type in appearance (B’), with a dorsal open phenotype (B’’), or with an anterior open phenotype (B’’’). Compared with *acal^5/5^* mutants, the cuticular phenotypes are the same, but they differ significantly in the abundance (expression of *acal* significantly reduces the number of mutant embryos that die and that have cuticular phenotypes). In these and following experiments, cuticular phenotypes do not change, unless otherwise stated. No new cuticular phenotypes are found; thus, examples are not depicted in all figures. Numbers analyzed: *acal^5/5^,UAS-acal/+* = 240, *acal^5/5^;69B-gal4/+* = 441, *acal^5/5^;69B>acal* = 237, *acal^5/5^;pnr-gal4/+* = 435, *acal^5/5^;pnr>acal* = 130. Statistical significance was calculated using chi square tests. (C) Quantification of in situ hybridization shown in (D) in stage 13 embryos (*acal^5/5^*, n = 10; *yw*, n = 6). *acal* mutants have significantly lower expression levels in lateral epithelia; chi square test. (D) *acal* in situ hybridization in embryos throughout embryonic development. Wild type embryos are *yw*, and mutant is *acal^5/5^*. Arrows indicate expression in the lateral epidermis and arrowheads point to expression in the central nervous system. Sense (negative) controls are also shown. Insets show boxed areas in stages 13, 15, and 17 embryos. See also S2 Fig.

**Figure 3:**
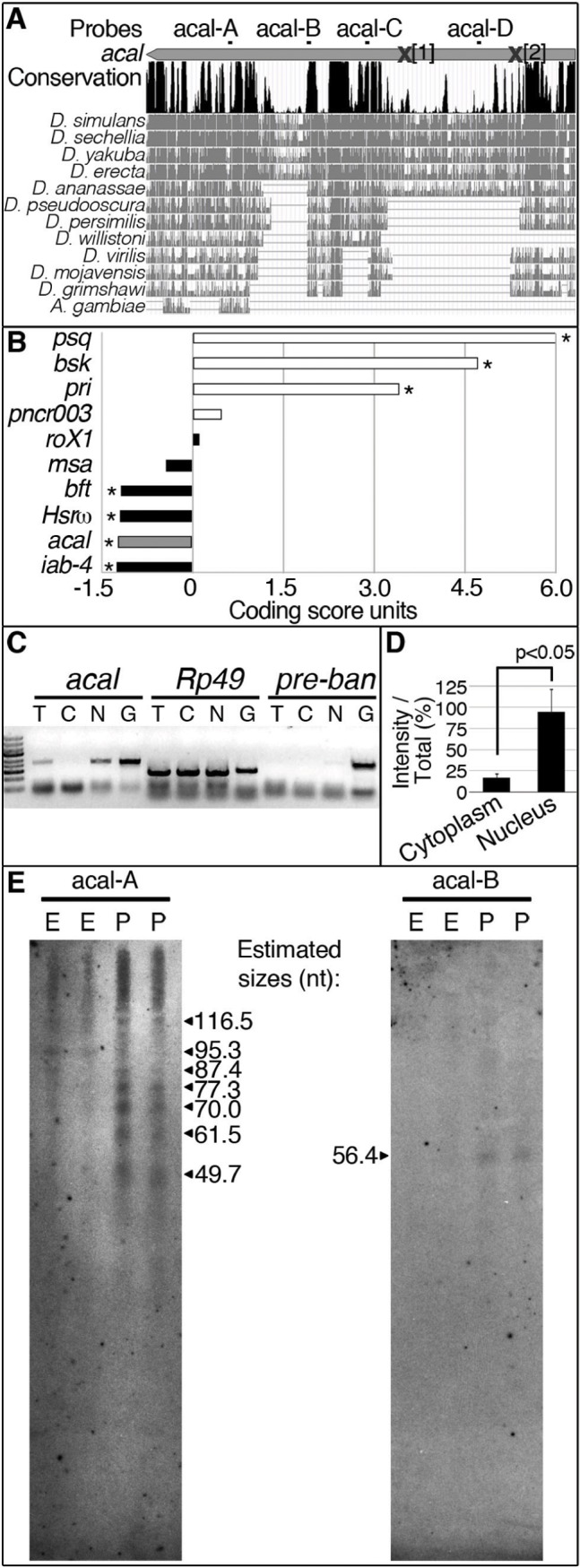
*acal* is a processed long non-coding RNA. (A) *acal* full-length transcript sequence conservation plot compared to other dipterans, and pairwise alignments between *acal* in *D. melanogaster* and its homologs (adapted from the UCSC genome browser). Location of molecular lesions in *acal^1^* (1) and *acal^2^*(2) are marked within brackets after an ‘**X’** in the transcript (gray arrow). Above the plot is the location of the probes used for small RNA Northern blots in (E) and in S3 Fig. (B) Protein coding potential of *acal* and other well known coding (white) and non-coding (black) RNAs. *pipsqueak (psq)* is a transcription factor and *basket (bsk)* codes for JNK. *polished rice (pri)* and *pncr003* are polycistronic and code for small peptides. *roX1* (*RNA on the X1*), *iab-4* (*infra-abdominal 4)*, and *Heat shock RNA ω* (*Hsrω)* are long non-coding RNAs. *bereft (bft)* is a microRNA precursor. Negative values correspond to non-coding scores, and positive values are for protein coding RNAs. Asterisks denote significantly coding or non-coding scores. (C) Semi-quantitative RT-PCR of total [T], cytoplasmic [C], and nuclear [N] RNA against *acal, Rp49* (protein coding gene), and *bantam* microRNA precursor (*pre-ban*). [G] is the genomic control. (D) Band intensity of *acal* cytoplasmic and nuclear amplification, compared to total RNA amplification. Means of 5 independent experiments +/− SEM. Significance was assessed using Student’s t test. (E) Small RNA Northern blots for *acal* A and B probes, using wild type embryos [E] and pupae [P] RNA. Sizes were estimated from 4 independent experiments.

We sequenced 4.7 kb of the SD08925 genomic region in *acal* mutant and control lines. In these and following experiments, we selected and separated mutant embryos from heterozygous controls by lack of embryonic GFP-expression due to a balancer chromosome (see Materials and Methods). Within this region, we found a 20 base pair insertion in *acal^1^*. The *acal^1^* lesion is 1031 bp away from *P{KG09113}*, at +931 of the cDNA, a position conserved in closely related species. Similarly, we found a 2 bp deletion 1630 bp away from *P{KG09113}* within the 5’ end of SD08925 in *acal^2^*, in a conserved region. In *acal^5^* SD08925 expression is significantly reduced(Fig. 1M’’, Fig. 2A and D and 3A).

In *acal^1^* and *acal^2^*, no trace of the original P element was found. We reasoned that both lesions, found 1–2 Kb away from the original insertion site (which is in itself viable and fertile), are likely consequences of P-element imprecise excisions and repair, due to nicking and repair of the DNA break, leading to lethality, and responsible for the *acal^1^* and *acal^2^* mutant phenotypes, respectively. In both cases, the changes are not present in any of the other strains (genome reference assembly, *yw* wild type control, parental strain, or other *acal* mutants). *acal^6^* is a P-element insertion mapping to the locus, 3 Kb 5’ from the transcription start site. We found no differences in the sequence in the 4.7 kb genomic region sequenced centered around the SD08925 transcribed region for *acal^3^*, *acal^4^*, and *acal^5^*. The sequenced genomic region includes the whole transcript, plus some neighboring 5’ and 3’ sequences (Fig. 1M’’), suggesting that mutations in *acal^3^*, *acal^4^*, and *acal^5^* lie outside the transcript. This is consistent with a regulatory nature for these alleles, rather than molecular lesions within the transcribed region, like *acal^6^*.

We performed a genomic rescue experiment (Fig. 1M’–N), rescuing fully the embryonic lethality of *acal^1^* and *acal^2^* mutants with a ∼20 kb genomic construct called *178D09. 178D09* spans a small portion of the 5’ region of *lola*, wholly the SD08925 transcript region, plus ∼7 kb upstream of SD08925 in the intergenic region between SD08925 and *psq* (Fig. 1M’). Besides the 5’-most exon of *lola*, which is part of the untranslated 5’ leader sequence of some *lola* splice variants and *CR45135*, no other transcript is included, except SD08925. SD08925 expression levels are not changed in rescued embryos versus controls, presumably due to endogenous regulatory sequences maintaining overall low expression levels (S1E Fig.). SD08925 is conserved in eleven Drosophila species surveyed, and recognizable in *A. gambiae* (Fig. 1O; 3A). Together, these data are consistent with a low-expression locus involved in DC.

We quantified SD08925 expression during DC in *acal^2^* and *acal^5^* mutant embryos by qRT-PCR. *acal^2^* expression is similar to wild type, but, as stated, *acal^5^* expression is significantly reduced (Fig. 2A). This is consistent with *acal^5^* being a regulatory mutant, although other interpretations are possible. To ensure consistency, whenever possible we performed experiments with multiple *acal* mutant alleles, with similar results. These data are also consistent with SD08925 coding for *acal*. In contrast, using primers that detect all *lola* isoforms, or all *psq* isoforms (both loci have alternative splicing) no significant differences in expression levels are seen in *acal^1^*, *acal^2^*, *acal^5^*, and *acal^6^* (S7C Fig.).

In order to more rigorously test whether SD08925 is *acal*, we generated a UAS transgene containing only the complete SD08925 cDNA (2.3 kb). We expressed this construct in *acal^5^* mutants, using the *69B* ectodermal driver. This resulted in significant rescue of *acal^5^* DC defects and reduction of embryonic lethality (Fig. 2B–B’’’). *69B* over-expression of *UAS-acal* in *acal^5^* mutants significantly increased SD08925 expression (40%, S2A Fig.). We also show that *pnr^MD237^* *gal4* (*pnr-gal4*) driven expression of SD08925 rescues DC defects in *acal^5^* (Fig. 2B). Regardless of the nature of *acal^5^*, augmenting SD08925 expression in the mutant significantly rescues the embryonic phenotypes, arguing that reduction of *acal* expression is responsible for the *acal^5^* DC and embryonic lethality phenotypes. Taken together, (1) the complete lack of complementation to each other and similar mutant phenotypes of all six *acal* alleles (but complementation to and dissimilar to mutant phenotypes of the neighboring *lola* and *psq* loci), (2) the molecular mapping of *acal^1^*, *acal^2^*, and *acal^6^* to the SD08925 locus, (3) the significant genomic and UAS-*acal* rescues for *acal^1^*, *acal^2^*, and *acal^5^*, and (4) the significantly reduced SD08925 expression in *acal^5^* both by qRT-PCR and *in situ* hybridization (see below), compared to no differences in *lola* and *psq* expression levels in *acal^1^*, *acal^2^*, *acal^5^*, and *acal^6^* establish the SD08925 locus as the *acal* locus.

### *acal* embryonic expression

In situ hybridization with SD08925 in wild type embryos revealed a dynamic expression pattern, mainly in two tissues: the lateral epidermis and the central nervous system. At early stages, the transcript is detectable throughout the embryo but falls at gastrulation (Fig. 2D). From germband retraction to DC, expression is seen in germband derivatives, mainly the lateral epidermis and nervous system (Fig. 2D). This is consistent with a role in DC and the *UAS-acal* rescue in the ectoderm and/or lateral epidermis (Fig. 2B). Later, expression appears in the mesoderm, but the bulk of expression is in the condensing nervous system and closing lateral epithelia. At the end of embryogenesis, expression occurs in the nervous system (Fig. 2D).

In *acal^5^* mutant embryos, expression is significantly reduced in the lateral epithelium during DC, consistent with *acal^5^* being a regulatory mutant and with the UAS rescue (Fig. 2B–D). Nervous system expression is not altered in *acal^5^* during DC stages, but decreases significantly later (S2B–C Fig.).

### *acal* is a processed, long non-coding RNA

Is SD08925, from now on referred to as the *acal* transcript, translated? The *acal* transcript is 2.3 kb long and could potentially code for protein(s). The locus is also evolutionarily conserved in other dipteran species (Fig. 3A), but not outside Diptera.

We find that in the dipteran species examined, the locus is devoid of conserved open reading frames (ORFs), sporting only variable, non-conserved ORFs ranging from 33 up to 243 nucleotides in size (S3A Fig.), even though other regions of the transcript are conserved (Fig. 3A; S3B Fig.). Unlike short ORF-bearing transcripts like *polished rice* (*pri*, [28]), the putative short ORFs in SD08925 and homologues have no common motifs. The deduced protein sequences of these ORFs in *D. melanogaster* show no homology with annotated proteins, and are not present in Drosophila proteomics databases (see Materials and Methods). Using an algorithm that measures coding potential [29], based on six different criteria [(1) feasibility of ORFs, (2) coverage of ORFs within transcript, (3) presence of in-frame initiation and stop codons, (4) number of hits in BLASTX, (5) high quality in any given BLASTX hit, and (6) whether any given hit is in frame with the predicted ORFs], we determined that *acal* has an extremely low coding potential, similar to other characterized long non-coding RNAs (Fig. 3B).

Genes harboring short ORFs have been erroneously classified as long non-coding RNAs, and later found to code for small peptides [28,30,31]. Does *acal* code for short, translated ORFs? Genes coding for short ORFs still have a protein coding score, unlike *acal* (Fig. 3B), and are exported to the cytoplasm for translation. In contrast, many non-coding RNAs reside in the nucleus. We detected *acal* transcripts enrichment in a nuclear fraction, different from *Rp49*, a protein-coding mRNA (Fig. 3C–D). As control, we also detected a small enrichment in the nuclear fraction of *pre-bantam (ban)*, a well-known miRNA precursor. The enrichment is small, likely because it is quickly processed to mature *ban. acal* nuclear enrichment also suggests a non-coding nature.

High-throughput RNA sequencing projects have deeply annotated small RNAs in the Drosophila genome [32]. Within *acal*, we found two groups of sequence reads reported to appear under various experimental conditions that did not qualify as functional small RNAs. We designated these groups of reads as acal-A and acal-C (Fig. 3A), and performed small RNA Northern blotting, reasoning these could be the mature products of the *acal* transcript. With this Northern protocol, larger RNA fragments are not detected, but small RNA species are well separated. Using the acal-A probe, we found evidence of *acal* fragmentation throughout the life cycle of the fly, particularly during pupal stages (Fig. 3E; S3C). Instead of a roughly 22-nucleotide fragment, we found a group of small bands from about 50 to 118 nucleotides (Fig. 3E). These bands are not detectable in regular agarose gels due to their size. In contrast, acal-C did not reveal evidence of processing (S3C Fig.). We used two additional probes, acal-B and acal-D, to look for evidence of further processing. acal-D revealed no processing (S3C Fig.), but acal-B revealed at least a 59 nucleotides-long fragment (Fig. 3E). Both acal-A and acal-B fall within conserved positions of the gene at the 3’ end (Fig. 3A); acal-B lies within a 60 nucleotide-long conserved region, a size coincidental with the band we found in small RNA Northern blots (for detail, see S3B Fig.). In conclusion, we have evidence that the 3’ half of the *acal* transcript undergoes processing, whereas the 5’ half does not. Unfortunately, as the signal we detected was mostly from pupae, we could not test mutants, because mutants die as embryos and larvae, and never reach pupation proper. Taken together, we propose *acal* is an unconventional long non-coding RNA partially processed into small fragments.

### *acal* down-regulates JNK signaling during DC

**Figure 4:**
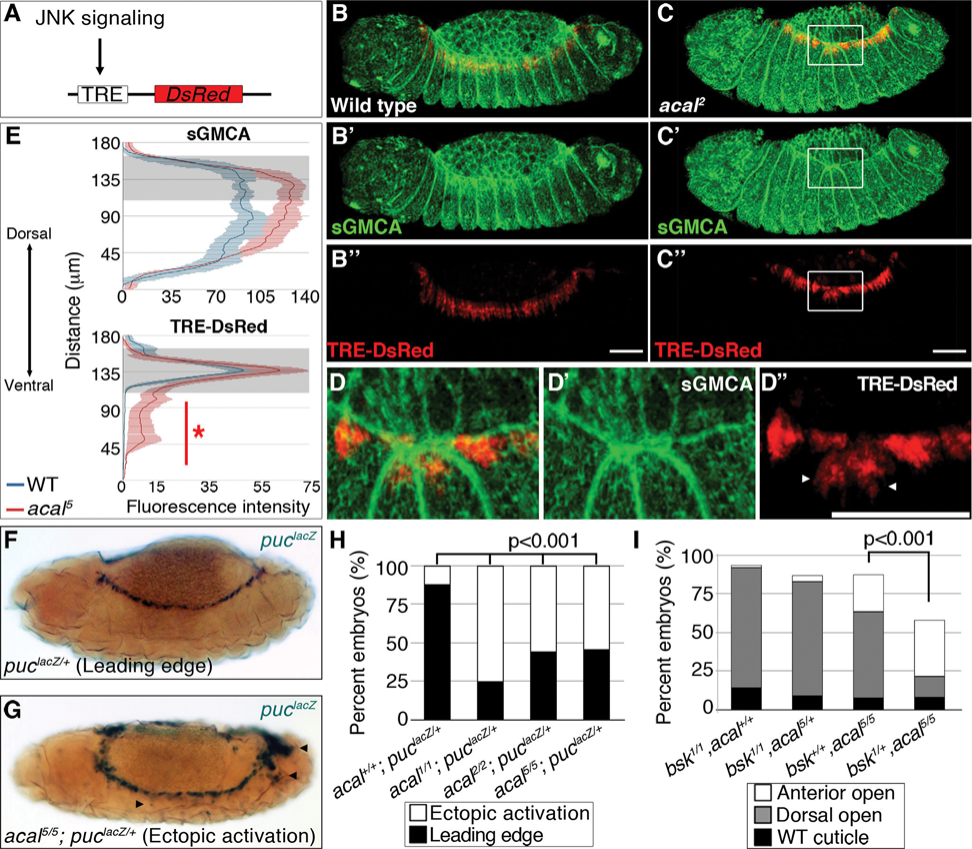
Ectopic JNK signaling activation in *acal* mutants. (A) TRE-DsRed works as an AP-1 responsive element driving expression of DsRed. (B–B’’) Wild type embryos, (C–D’’) *acal^2^* mutants. sGMCA is a marker for cortical cytoskeleton (green in B, C, and D). (B–D) Images are representative of five embryos per condition. (D–D’’) Boxed area in C. Ectopic TRE-DsRed activity is shown (arrowheads). Scale bars in (B, C, and D) are 50 μm. (E) Mean fluorescence intensity +/− SEM of sGMCA and TRE-DsRed in *acal^5^* mutants and in control embryos. Leading edge region is highlighted in gray. n = 5 for each condition. For both channels, distributions are significantly different (Kolmogorov-Smirnov test, asterisk and bar depicting significant ectopic JNK activation in lateral epithelia ventral to the LE, p<0.001). (F-H) *puc^lacZ^* staining of control (F) and mutant (G) embryos. Arrowheads in (G) mark ectopic *puc^lacZ^* activation. (H) Quantification of *acal* mutant phenotypes using *puc^lacZ^* heterozygosity as JNK signaling reporter and genetic sensitized background. Mutant embryos were selected by lack of *eve-lacZ* staining, present in balancer chromosomes. Number of embryos analyzed: *acal^+/+^;puc^lacZ/+^* = 108, *acal^1/1^;puc^lacZ/+^* = 229, *acal^2/2^;puc^lacZ/+^* = 48, *acal^5/5^;puc^lacZ/+^* = 62. See also S4 Fig. (I) Genetic interaction between *bsk^1^* and *acal^5^* mutants. Only the dead embryos are classified, the mutants surviving embryogenesis are the remaining percentage to amount to a hundred percent (open space above bars). Number of animals analyzed: *bsk^1/1^;acal^+/+^* = 154, *bsk^1/1^;acal^5/+^* = 210, *bsk^+/+^;acal^5/5^* = 391, *bsk^1/+^;acal^5/5^* = 166. Significance in (H-I) was calculated using chi square tests.

We next studied JNK signaling, the DC trigger, to characterize DC defects in *acal*. We tracked and quantitated expression of a DsRed reporter under control of an AP-1 response element, TRE (Tetradecanoylphorbol acetate Response Element, see Fig. 4A and [33]). In parallel, we tagged cellular membranes using sGMCA, a fusion protein of the actin-binding domain of Moesin and GFP [34]. These constructs were expressed in an *acal* mutant and wild type backgrounds. In stage 13 wild type embryos, TRE-DsRed marks only the LE [a very faint amnioserosa DsRed positive staining is also present (Fig. 4B, 4B’’)]. At this stage, lateral epidermal cells are stretching towards the dorsal side (Fig. 4B, B’). In *acal* mutants, the TRE-DsRed is ectopically expressed in the lateral epidermis and in some amnioserosa cells (Fig. 4C–E). Quantitation of the signal uncovers significantly higher JNK activation levels in the lateral epidermis of *acal* mutants, and a significantly higher sGMCA signal, consistent with the observed mis-stretching and local accumulation of lateral epithelial cells at the border of the epithelium during DC (Fig. 4E). Ectopic JNK activation coincides with mis-stretching of the lateral epidermis, seen as a contraction of the epithelium where JNK ectopic activation occurs (Fig. 4C–D). Similar results are seen at later DC stages (S4A–D Fig.). JNK ectopic activation in the amnioserosa of *acal* mutants is likely a consequence of lateral epithelium defects, as *acal* rescue solely in the lateral epithelium rescues DC defects. Significant ectopic JNK reporter activity in the lateral epidermis underneath the leading edge cells and in the amnioserosa was also observed using another weaker *acal* allele (*acal^2^*) (S4E–G Fig.), with a GFP-based TRE JNK activity reporter [33]. Results for *acal^2^* are normalized for nuclear density. This strongly suggests that *acal* DC defects are due to JNK gain-of-function signaling in more lateral epidermal cells, resulting in disorganized stretching.

As a second and independent means to study the consequences of *acal* mutations in JNK activity, we over-activated JNK signaling in *acal* mutant and control embryos by expressing both a constitutively active form of the JNK kinase, Hep (Hep^ACT^), and a wild type version of the same gene (Hep) in the ectoderm (using *69B-gal4*). We found that expressing *hep^ACT^* in *acal* heterozygotes results in significantly stronger and more penetrant closure defects compared to wild type embryos expressing *hep^ACT^* (S4I Fig.), exacerbating the *hep^ACT^* over-expression phenotypes and supporting the notion that *acal* mutants promote increased JNK activity. We also see the appearance of a new phenotype, ‘early’ embryonic death, where embryos die without forming a cuticle. Over-expression of *hep* in *acal* heterozygotes has milder effects, but significantly leads to an increase in death of fully formed embryos in *acal* heterozygotes, compared to wild type embryos over-expressing Hep (S4H Fig.). Homozygosity for *acal^5^* together with *hep^ACT^* over-expression, or for *acal^2^* together with *hep* over-expression, lead to significant further increases of the respective mutant phenotypes (S4H-I Fig.). These *hep* gain-of-function experiments together with *acal* loss-of-functions are all consistent with a role for *acal* in negative regulation of JNK activity during dorsal closure in the lateral epithelium.

We then corroborated this in two other independent ways: using a second JNK signaling reporter, and performing genetic interactions with JNK mutants. If *acal* acts to negatively regulate JNK activity, then in *acal* mutants we should see enhanced JNK reporter line expression (similar to the TRE results, above), or suppression of *acal* phenotypes in partial JNK loss-of-function.

*puc* is a transcriptional target of JNK signaling coding for a JNK phosphatase, a negative feedback loop that halts JNK. *puc^E69^* is a *lacZ* enhancer trap allele (from now on referred to as *puc^lacZ^*). Heterozygosity for *puc^lacZ^* provides a JNK sensitive signaling reporter, and a sensitized background disrupting the feedback loop [18]. Despite decreased *puc* dosage in *puc^lacZ^* heterozygotes, in approximately 90% of embryos, JNK signaling is restricted to LE (Fig. 4F–H). In *acal* homozygotes (*acal^1^, acal^2^*, or *acal^5^*), heterozygous for *puc^lacZ^*, the proportion of embryos with ectopic *puc^lacZ^* expression in amnioserosa and lateral epidermis significantly grows to around 50% or more, similar to JNK ectopic activation with TRE-constructs (Figs. 4G–H and S4A–G).

*acal* mutants heterozygous for *puc^lacZ^* also show more extreme cuticle phenotypes. This is consistent with a potentiation of the *puc* effect on *acal* phenotypes, both genes acting to counteract JNK signaling (S5A Fig.).

Finally, we reduced JNK dosage to see whether it would ameliorate *acal* mutant phenotypes. Heterozygosity for *bsk^1^*, a JNK loss-of-function mutation, significantly suppresses *acal^5^* phenotypes (Fig. 4I), meaning that JNK signaling over-activation of *acal* mutants depends on JNK itself. Conversely, *acal^5^* heterozygosity does not alter the *bsk^1^* mutant phenotype (Fig. 4I), consistent with *acal* mutant phenotypes dependent on JNK. We confirmed this genetic interaction in a different genetic background and found similar results (S5B Fig.). Taken all data together, the (1) two types of JNK reporters (TRE-based and *puc^lacZ^* ) tested with several *acal* alleles, and (2) genetic interactions for several *acal* alleles with *bsk* and *puc* (loss-of-function), and *hep* (ectopic expression), all establish *acal* as a negative regulator of JNK signaling.

We next tested if *acal* mutations interact with dorsal open group mutations known to act in the amnioserosa, in order to determine if *acal* could have a function in this tissue. We used *peb^308^*, a hypomorphic mutation that affects *peb* expression in the amnioserosa, and, consequently, DC [19]. The *peb^308^* DC defects were not modified in an *acal* sensitized background, consistent with *acal* required only in the lateral epidermis (S5C Fig.).

### The pioneer protein Raw and *acal* act together to counteract JNK signaling

**Figure 5:**
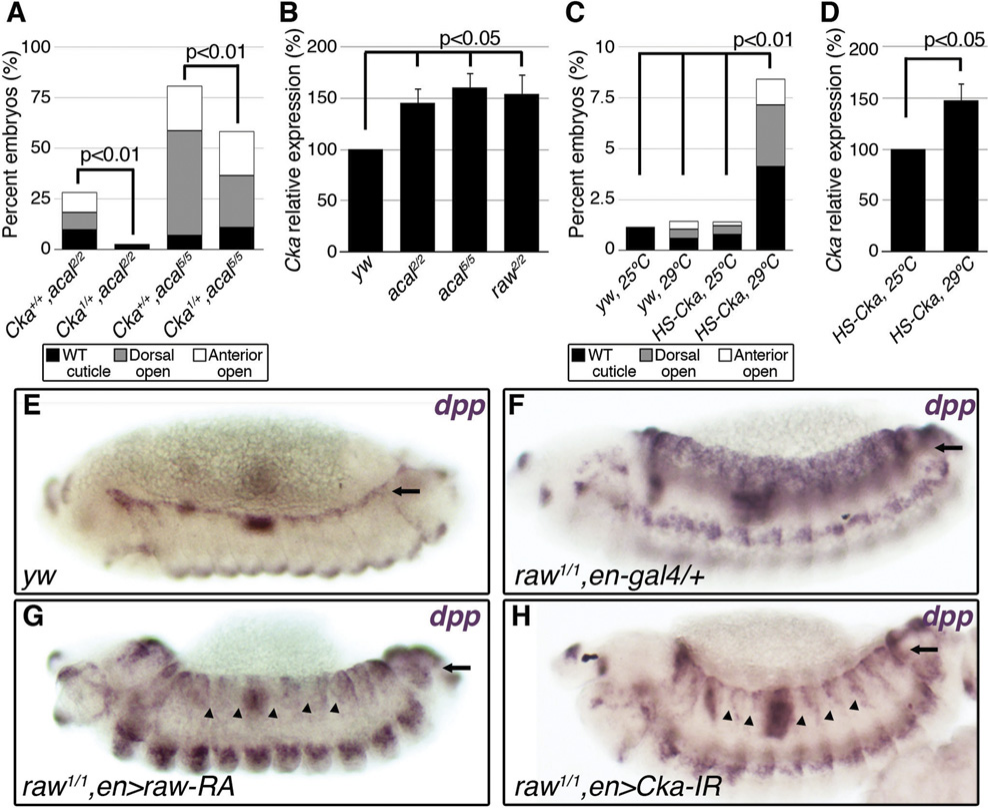
*raw* and *acal* act together to counteract JNK signaling. (A) Wild type cuticle. (B) Cuticle phenotype of *raw* mutant embryo. (C) Genetic interaction between *raw^2^* and *acal^5^* mutants. *raw*-like phenotype is depicted in (B). In *raw^+/+^; acal^5/5^* mutants a small percentage survives embryogenesis, and constitute the open space above the bar to amount to a hundred percent total. Number of animals analyzed: *raw^+/+^,acal^5/5^* = 391, *raw^2/+^,acal^5/5^* = 139, *raw^2/2^,acal^+/+^* = 366, *raw^2/2^,acal^5/+^* = 152, *raw^2/2^,acal^5/5^* = 208. Significance was assessed with chi square tests. (D-E) *acal* in situ hybridization in *raw^2^* mutants (n = 45; D) and heterozygous siblings (n = 106; E). Arrow in (E) points to decreased *acal* expression in the lateral epidermis. See also S5 Fig. (F-I) Scanning electron micrographs of dorsal views of adult thoraces, anterior is up. Scale bars are 100 μm. (F) *UAS-acal/+* control, (G) *pnr-gal4/+* control, and (H) over-expression of two *UAS-acal* copies. The white box in (H) is amplified in (I), depicting distances (red lines) measured to determine the thoracic cleft index, using anterior dorso-central (ADC) and posterior dorso-central (PDC) bristles as references (see Materials and Methods). (J) Percentage change of thoracic cleft index for different experimental conditions. Mean of 15 flies +/− SEM. Significance was calculated using ANOVA and Bonferroni correction.

**Figure 6:**
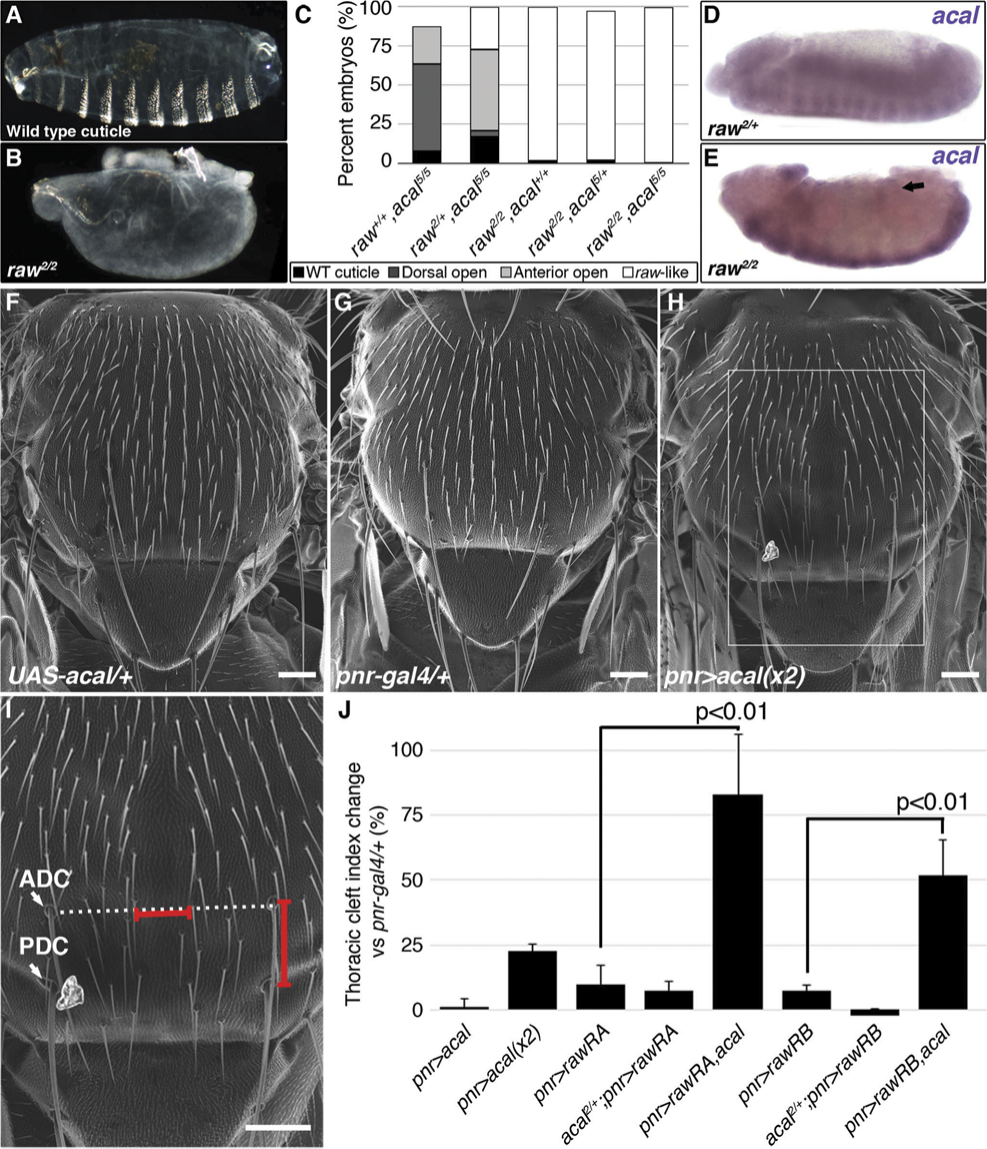
*Cka* is downstream of *raw* and *acal*. (A) Genetic interaction between *Cka* and *acal* mutants. Number of animals analyzed: *Cka^+/+^,acal^2/2^* = 192, *Cka^1/+^,acal^2/2^* = 219, *Cka^+/+^,acal^5/5^* = 391, *Cka^1/+^,acal^5/5^* = 171. (B) *Cka* relative expression in wild type and mutant embryos, as determined by qPCR. (C) Expression of a heat-shock inducible *Cka* transgene results in DC defects. Number of animals analyzed: *yw*, 25°C = 526, *yw*, 29°C = 591, *HS-Cka*, 25°C = 2006, *HS-Cka*, 29°C = 3227. (D) *Cka* expression increase due to heat shock in *hs*-Cka flies was confirmed by qPCR. For (A,C) embryos surviving embryogenesis represent the open space above the bar to amount to one hundred percent total of embryos analyzed. Chi square tests were used to calculate significance. For (B,D) represents the means of three independent experiments run twice +/− SEM. Significance was assessed using Student’s t test. (E-H) *dpp* in situ hybridization experiments, showing JNK-induced *dpp* expression (arrows). (E) Wild type embryo. (F) *raw^1/1^,en-gal4/+* control, showing *dpp* ectopic activation (arrow). (G) Expression of *UAS-rawRA* with *en-gal 4*, which expresses *gal 4* at posterior compartments of each segment. Arrowheads show cell-autonomous suppression of *dpp* ectopic expression. (H) Silencing of *Cka* with an RNAi construct (*UAS-Cka-IR*) under *en-gal 4* also suppresses *dpp* ectopic expression in posterior compartments of *raw^1^* mutants (arrowheads).

*acal* might interact with other negative regulators of JNK signaling. *raw*, a conserved gene of unknown molecular function, counters JNK signaling in the lateral epithelium, like *acal. raw* and *acal* have very similar expression patterns during DC (Fig. 2D for *acal*; for *raw* see [17]). Raw function is genetically positioned at the level of JNK [35], like *acal*, and Jra [17]. *raw* mutant cuticular phenotypes are stronger than *acal*, with dorsalized embryos consequence of *dpp* ectopic expression at the lateral epidermis (see Fig. 5A–B and Fig. 6E–F, [17]).

*raw* mutants have *dpp* ectopic expression during DC in rows of cells ventral to the leading edge ([17] and S6C Fig.). *acal* ectopic expression in the posterior part of each segment of these embryos (*en-gal4* driver) reduces cell-autonomously *dpp* over expression (S6D Fig.). This is consistent with *acal* acting to inhibit JNK pathway on the lateral epithelium during DC. On their own, *acal* mutant embryos are not dorsalized (Fig. 1E-L), and show no clear ectopic *dpp* over-expression (S6A–B Fig.), yet at least under conditions of *dpp* over-expression, *acal* can repress *dpp*. Altogether, this argues that *acal* is a negative regulator of JNK activity, and that it has a subtler effect to that of *raw*.

We analysed genetic interactions between *acal* and *raw*. Heterozygosity for *raw^2^* increases the embryonic lethality of *acal^5^* embryos, with a quarter phenocopying the *raw^2^* dorsalized phenotype (‘*raw*-like phenotype’, Fig. 5C). *acal^5^* heterozygosity does not enhance the already strong *raw^2^* homozygous condition, and double homozygotes do not show a stronger phenotype. Similar results are observed between *acal^2^* and *raw^1^* (S6E Fig.). These genetic interactions are consistent with *acal* acting downstream of *raw*.

We looked for *acal* expression in *raw* mutants, and found that *acal* lateral epidermis expression is significantly decreased in these embryos (Fig. 5D–E). This suggests that *raw* regulates *acal* lateral epidermis expression. Nervous system *acal* expression is unchanged in these *raw* mutant embryos, acting as an internal control. This also ties in with *acal^5^* being a regulatory mutant with reduced lateral epithelium expression (Fig. 2D), and suggests a *raw* dependent *acal* control module for the lateral epidermis. Consistent with *acal* acting downstream of *raw*, as a *raw* effector, *69B-gal4 UAS-acal* expression partially rescues the *raw* dorsalized cuticle phenotypes (S6F–H Fig.), as expected from the *dpp* in situ hybridization in *raw* mutants over-expressing *acal* (S6D Fig.). Altogether, these results indicate that *acal* mediates part of Raw function during DC.

*raw* over-expression in the lateral epidermis of wild type embryos has no effect in DC, in spite of the strong *raw* loss-of-function phenotype [17]. Likewise, *acal* over-expression in wild type embryos has no DC effects (embryonic lethality of *69B>acal* embryos is around 1%, n = 791; also *pnr^MD237^>acal* shows no lethality or overt phenotypes, with equal numbers of control and over-expression siblings, n = 104). These lack of gain-of-function phenotypes for both genes indicate that *raw/acal* JNK negative regulation has a limit beyond which it cannot be exercised further during dorsal closure, suggesting other regulatory mechanisms might act in concert.

Thoracic closure during metamorphosis also depends on JNK signaling, much like DC does [36,37] Altering JNK signaling at the notum anlagen results in an antero-posterior cleft spanning the adult thorax (Fig. 5G–H). Some JNK signaling loss-of-function conditions have cleft thoraces [38,39]. This process has been used also in miss-expression screens for JNK signaling, where gain-of-function phenotypes are repeatedly encountered [40]. We thus used thorax closure as a model where we might be able to study *acal* and *raw* gain-of-function conditions. For this, we hypothesized that *acal* and/or *raw* over-expression at the notum anlagen could result in a cleft phenotype, using *pnr-gal4* (expressed at the dorsal heminota, equivalent to the lateral epidermis, Fig. 5F–H). *pnr-gal4* also provides a sensitized genetic background, as heterozygosity for this allele generates a mild thoracic phenotype, increasing the sensitivity of the assay [41].

Thoracic cleft was not enhanced by *raw* notum over-expression, as quantified by a ‘Thoracic cleft’ index (Fig. 5I-J; Materials and Methods). Similar results were observed for RawRA or RawRB isoforms, generated by alternative splicing (Fig. 5J). In contrast, two copies of the *UAS-acal* transgene had a significant effect (one copy was ineffective; Fig. 5H–J). If *raw* over-expression induces *acal*, adding more *acal* would then lead to a more extreme phenotype, as adding two transgene copies of *acal* do in the dorsal thorax. Simultaneous over-expression of *acal* and *raw* single copy transgenes had a significant synergistic effect, inducing stronger cleft formation (Fig. 5J). Both *raw* isoforms show synergism with *acal*. This is consistent with *raw* positively regulating *acal*, and both counteracting JNK signaling. Taken together, these results show that *acal* and Raw act together to counteract JNK signaling, *acal* downstream of *raw*. This is consistent with Raw acting at the level of JNK and/or Jra, as our experiments with *acal* point.

### Expression of *Cka*, a JNK scaffold, is regulated by Raw and *acal*

**Figure 7:**
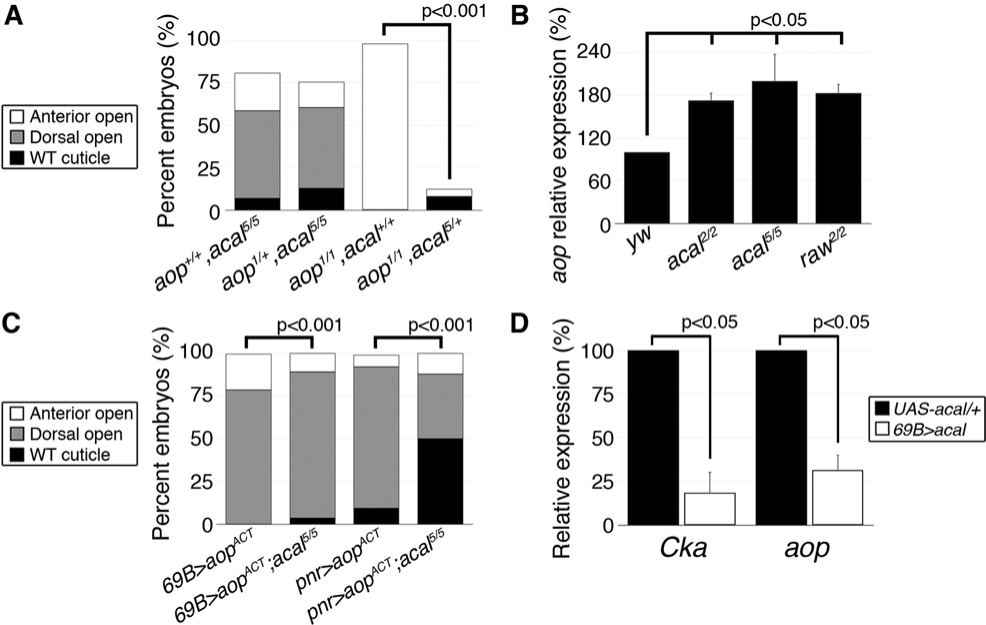
*acal* regulates *aop* expression. (A) Genetic interaction between *aop* and *acal* mutants. Embryos surviving embryogenesis represent the open space above bars to amount to a hundred percent total embryos analyzed. Number of animals analyzed: *aop^+/+^,acal^5/5^* = 391, *aop^1/+^,acal^5/5^* = 253, *aop^1/1^,acal^+/+^* = 269, *aop^1/1^,acal^5/+^* = 185. (B) Relative expression of *aop* in wild type and mutant embryos, as determined by qPCR. (C) Expression of a constitutive active *aop* transgene (UAS-*aop^ACT^*) in the ectoderm (*69B-gal4*) or in the lateral epidermis (*pnr-gal4*), of wild type or *acal* mutant embryos. Number of animals analyzed: *69B>aop^ACT^* = 642, *69B>aop^ACT^;acal^5/5^* = 210, *pnr>aop^ACT^* = 310, *pnr>aop^ACT^;acal^5/5^* = 180. (D) *acal* over-expression under the *69B-gal4* driver results in significantly decreased *Cka* and *aop* expression. In (B) and (D), graphs show mean values +/− SEM of at least three independent experiments run twice.

Both *raw* and *acal* act genetically at the level of JNK (Fig. 4I; [35]). Yet *raw* has been shown to act also at the level of Jra [17]. Could *raw* do both? Could *raw* via *acal* modulate a mediator of both JNK and Jra, for example? Cka is a scaffold protein that interacts with JNK and Jra [20]. We tested whether *acal* and *Cka* interact genetically. *acal* DC defects are dependent on *Cka*, since heterozygosity for *Cka^1^* rescues the *acal* mutant phenotype (Fig. 6A). This is in fact a stronger genetic interaction than that seen with *bsk* (JNK) mutants. Moreover, *Cka* expression is similarly significantly increased (to 50% above control values) in both *acal* and *raw* mutants (Fig. 6B). Conversely, *acal* embryonic over-expression (gain-of-function) leads to a significant reduction in *Cka* expression in otherwise wild type embryos, although this condition bears no observable phenotypic consequences (Fig. 7D).

To test the *raw-acal-Cka* genetic pathway, we silenced *Cka* to see if it suffices to rescue the *raw* mutant phenotype, using again the strong *raw* mutation-dependent *dpp* over-expression phenotype as readout (Fig. 6E–F). If this pathway is true, expressing *Cka* RNAi in the *en-gal4* expression pattern in *raw* mutants would silence *dpp* expression only at posterior compartments of each segment, the anterior compartments serving as internal controls. We found that *Cka* knockdown suppresses cell-autonomously *raw* ectopic *dpp* expression (Fig. 6F versus 6H), similar to rescuing *raw* function with *UAS-rawRA* (Fig. 6F–G). Taken together, both the loss-of-function as well as the ectopic expression results argue in favor of *raw* acting through *acal* down-regulating JNK signaling by regulating *Cka* expression, and accommodates the apparently conflicting results that *raw* acts at the JNK and/or Jun levels. Genetic interactions do not imply direct interactions, and so, *acal*, as a *raw* mediator, could act indirectly on *Cka* and regulate in this way JNK and Jra.

*Cka* sole over-expression should alter DC. Using a heat-shock inducible *Cka, hs-Cka* [20], we found that 29°C treatment of embryos throughout development significantly induces DC defects (Fig. 6C). Heat-shock induction indeed increases *Cka* expression (Fig. 6D). Taken together, these results define a novel regulatory path for JNK signaling, *raw* counteracting *Cka* expression through *acal*.

### *acal* fine-tunes DC by regulating *aop* expression

Does *acal* interact with other DC regulators? Aop serves at least two functions during DC. In the lateral epidermal cells, Aop promotes tissue differentiation by preventing ectopic mitoses. As an extension, in the LE, it has a second function preventing ectopic and precocious expression of JNK activity and of AP-1 target genes, and thus, acting against dorsal closure, an activity countered by JNK via phosphorylation. Hence, over-expression of a constitutively active Aop (*aop^ACT^*) that cannot be inactivated by JNK in the LE, results in DC defects due to lack of JNK targets expression. On the other hand, *aop* loss-of-function leads to anterior holes in the cuticle and faulty DC resulting from both ectopic mitoses in the tissue, and JNK over-activation. The wild type stretching lateral epithelium is a post-mitotic, competent tissue for DC in part due to *aop* dual roles (i.e., mitosis and precocious DC repression [15]). So, *aop* regulation can be a nodal, sensitive point for DC regulation. Genetic analyses show that *acal* and *aop* interact. *acal* mutations powerfully rescue *aop* loss-of-function, but *aop* loss-of-function does not alter *acal* mutants, showing that *acal* acts above or at the level of *aop* (Fig. 7A). Indeed, we found that *aop* expression is significantly increased in *acal* and *raw* mutants (Fig. 7B). Conversely, *acal* over-expression in wild type embryos leads to a significant reduction of *aop* expression (Fig. 7D), without phenotypic consequences (similar to *Cka*), consistent with a modulatory role for *acal*.

In accordance with an *acal* role as JNK negative regulator, *aop^ACT^* that presumably locks in the post-mitotic nature of the lateral epithelia, while preventing JNK target gene expression, is countered by *acal* mutants, more so if *aop^ACT^* expression is less widespread (Fig. 7C). A possible explanation for the puzzling interactions between *acal* and *aop*, resides in the dual nature of *aop* function in the LE and the lateral epidermis. In wild type embryos, *aop* is only phosphorylated and inactivated at the LE by JNK. Expressing *aop^ACT^* conceivably affects only the JNK-regulated function of *aop* (i.e., its role in the LE). Thus, the JNK over-activation seen in *acal* mutants might compensate partially for LE *aop^ACT^*. In contrast, in *aop* mutants, low *aop* levels throughout the epidermis might be partially restored to more wild type levels by *acal* loss-of-function conditions, as *acal* mutants over-express *aop*. Taken together, by regulating *aop* expression, *acal* can powerfully influence DC. In summary, we show that *acal* critically regulates both positive *(Cka)* and positive/negative *(aop)* components of JNK signaling.

### *acal* acts in trans during DC

How does *acal* regulate expression of other genes? One possibility is that *acal* acts in *cis*, exerting an effect on neighboring genes, something reported for other lncRNAs [10,41]. To study this possibility, we made use of a Gene Search insertion line, a transposon that can lead to ectopic expression outwards in both directions from the insertion side. *GS88A8* maps in the same interval as *acal*. Using *gal4* lines, *GS88A8* generates ectopic expression of neighboring genes, namely *lola* and *psq* [42]. We used the *69B-gal4* line to drive ectopic expression from *GS88A8* in the embryonic ectoderm. If *acal* negatively regulates neighboring genes *(lola* and *psq*) and they are relevant for DC, then ectopic expression from *GS88A8* should phenocopy *acal* mutant phenotypes. Instead, this condition mainly leads to larval and pupal lethality plus some adult escapers (S7B Fig.), with hardly any embryonic phenotypes, consistent with unaltered *lola* and *psq* expression profiles in *acal* mutants (S7C Fig.). Moreover, *acal* mutations complement *lola* and *psq* mutations (S1 Table), and mutations in these genes lead to different embryonic phenotypes (S7A Fig.). Altogether, we conclude that it is not very likely that *acal* acts in *cis* during DC.

Some long non-coding RNAs work in trans. One possibility is as part of chromatin remodeling complexes, for instance, although other mechanisms are possible [43,44]. In order to explore whether *acal* might function through remodeling complexes, we performed genetic interactions with *Polycomb^3^* (*Pc^3^*) mutants. Pc is a critical member of the Pc chromatin repressive complex 1 (PRC1, [45]). *acal* and *Pc^3^* double heterozygotes show novel strong wing defects [significantly twisted wings, with reduced posterior territory (S8A and F Fig.)]. Consistently, this interaction is seen with *raw* mutant alleles, albeit to a lesser degree (S8B Fig.). *Cka* and *aop* do not show this interaction (S8C–D Fig.).

*acal^5^* also significantly enhances the *Pc^3^* extra sex combs phenotype, as does *raw^1^*(*aop^1^* reduces extra sex combs, presumably due to a repressive function in sex combs formation, S8G–I Fig.). These non-allelic non-complementation results are reminiscent of the ‘extended-wing’ phenotype used to detect direct interactors / components of other chromatin remodeling complexes, like the *brahma* or *kismet* complexes [46], suggesting a common regulatory pathway for *acal*, downstream of *raw*, with PRC1. The fact that *raw* and *acal* show the same genetic interactions with *Pc*, but not *Cka* and *aop*, underscores the fact that *Cka* and *aop* are downstream from the former two (*raw* and *acal*), as regulatory targets in the same signaling cascade.

## Discussion

Several lines of evidence show that *acal* is a putative nuclear, processed, lncRNA, whose mutations are homozygous lethal, with requirements in DC. We show that *acal* mutants (three of which map molecularly to the SD08925, or *acal*, locus) fail to complement each other, but complement neighboring genes (*lola* and *psq*), regulate the same downstream genes (*Cka* and *aop*), have the same JNK negative regulation function [evidenced by two different reporter gene systems and genetic interactions with several JNK pathway component (*bsk, hep*, and *Cka*) and regulator genes (*puc, raw, and aop*)], are rescued by the SD08925 locus, with one allele having significantly reduced SD08925 expression, and all having similar mutant phenotypes and phenocritical periods. The locus produces a nuclear 2.3 Kb transcript with low coding potential that is processed into smaller pieces. Altogether, these data establish *acal* as a lncRNA locus in DC.

lncRNAs are ubiquitous species, probably tied to most biological processes. Yet, few have been characterized [47]. They fine-tune target abundance in multiple ways [1]. lncRNA species probably form diverse complexes with multiple mechanisms of action, and are not a rigorously defined class of molecules. Identifying lncRNA mutants with clear phenotypes in genetic models is a clear way forward. *acal* has an extremely low coding potential and no evolutionarily conserved ORFs. The putative *acal* ORFs show no homology to known peptides / proteins. Unlike short-ORF bearing transcripts [48], these putative ORFs have no sequence redundancy among them, reliable Kozak sequences, or high coding potential. This places *acal* as a non-translated transcript.

*acal* is a Dipteran-conserved locus. As many lncRNAs, it is not broadly conserved across species. *acal* RNA is processed into smaller fragments. These fragments have probably been missed in high-throughput sequencing projects due to RNA cut-off sizes [32]. *acal* functions in *trans* to regulate DC, as DC defects can be rescued in *trans* with a wild type full-length cDNA targeted to the epidermis.

*acal* foci for DC is the lateral epidermis. JNK loss-of-function result in lack or reduction of lateral epidermal cells stretching. In contrast, negative regulators, such as *peb, puc*, and *raw* also cause DC defects, due to disorganized and / or precocious cell stretching [13]. In *acal* mutants, cells also stretch in a disorganized manner. Predictably, reducing *bsk* gene dosage significantly reduces *acal* defects in DC, and JNK activity monitors show enhanced JNK function in *acal* mutants. This prompted study of *acal* and other negative regulators.

*raw* male gonad morphogenesis defects are ameliorated by *bsk* gene dosage reduction [35]. *acal* and *raw* collaborate to down-regulate JNK signaling at the lateral epidermis. In situ hybridization experiments, embryonic genetic interactions, and *raw* and *acal* over-expression studies in the thorax, put *raw* upstream of *acal*, yet they do not strictly phenocopy. This means that *raw* has other effectors. *raw* mutant embryos are dorsalized due to strong ectopic *dpp* expression in the lateral epidermis [17]. *acal* mutants do not dorsalize or show overt *dpp* misregulation. Furthermore, since Raw is localized in the cytoplasm, and *acal* is present in the nucleus, it is reasonable to assume that Raw regulates *acal* expression indirectly. Over-expression of either *raw* or *acal* in wild type embryos has no discernible effect on DC ([17] and this work), yet simultaneous over-expression in the thorax disrupts thorax closure, and *acal* over-expression reduces both *Cka* and *aop* transcript levels.

*Cka* expression is elevated in *raw* and *acal* mutants. Genetically decreasing *Cka* levels rescues DC defects in *raw* and *acal* mutants. Consistently, *Cka* over-expression itself causes DC defects. Work in the mammalian *Cka* functional homologue JIP-3 has shown this scaffold protein up-regulated under stress conditions. JIP-3 depletion and over-expression directly influence JNK activity [49]. Together with our results, these findings show that JNK scaffolds are an important point for JNK regulation, since augmenting scaffold availability augments JNK activity. In fact, this has been demonstrated biochemically for *Cka* [20].

Another critical JNK regulatory point by *acal* is *aop. aop* is also over-expressed in *acal* mutants, and down-regulated in *acal* over-expression. *aop* plays a complex role in DC making the tissue ready early, before DC, by preventing mitosis and early activation of JNK targets. For this, Aop function is required in the LE and lateral epidermal cells ventral to the LE [15]. Later, *aop* is inhibited by the JNK pathway only in the LE, allowing target gene expression there. In cells ventral to the LE, *acal* (together with *raw)* prevents JNK activation. In these cells there is no JNK activity to inhibit *aop*, yet *aop* expression regulation must be effected. This may be accomplished by *acal* (and *raw*). Thus, controlling *aop* levels, like *Cka* levels, may be a critical point for DC. For both genes, *acal* acts genetically as a repressor, with the net result of allowing JNK activity only in the LE in wild type.

**Figure 8:**
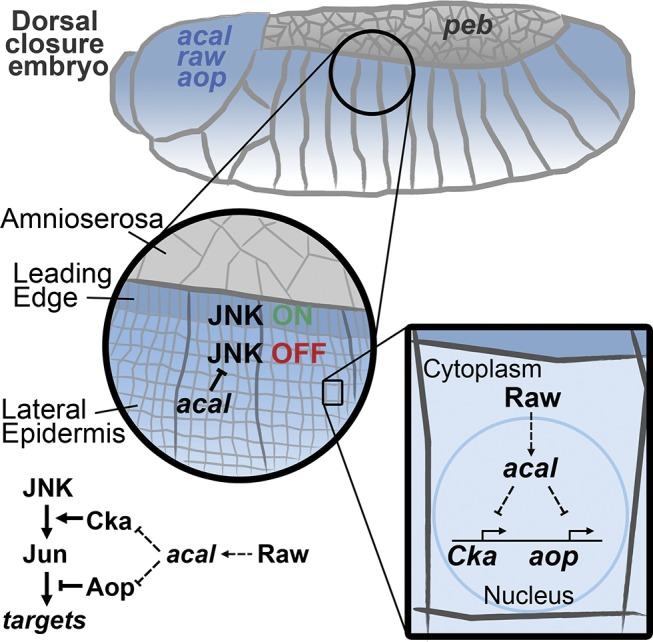
Model for *acal* function during DC. *acal*, expressed in the lateral epidermis (blue), restrains JNK activation to the leading edge cells. Raw, also expressed in the lateral epidermis, regulates *acal* expression. In turn, *acal* regulates the expression of *Cka* and *aop* to fine-tune JNK signaling. Not depicted is *aop* positive role during DC.

In *acal* mutants, *Cka* over-expression leads to JNK ectopic activation in more lateral epithelium cells. However, the fact that *aop* is also over-expressed in *acal* mutants may mask a stronger JNK over-activation, as JNK signaling may inhibit at least in part Aop function, Aop being a JNK direct target. Future work will focus on how *acal* fine-tunes these signaling genes (Fig. 8).

We show here that *acal* function modifies gene expression. Whether this is direct, as has been proposed for other lncRNAs [45,50], or is done in some other fashion, is not yet clear. *acal* interacts genetically with *Pc (raw*, that regulates *acal*, also interacts with *Pc)*. This opens the interesting possibility that *acal* acts directly with chromatin remodeling complexes, as has been demonstrated for other *Pc* interactors [42,51–53]. Yet other possibilities and/or indirect modes are clearly plausible.

In conclusion, we show that a lncRNA locus is required for DC regulation and JNK signaling. lncRNAs have not been implicated before in JNK signaling, and hence, this adds a new layer of control in JNK regulation.

## Materials and Methods

For detailed protocols, please refer to S1 Protocol.

### Fly husbandry

Detailed description of stocks is available in S1 Protocol. Crosses were done at 25°C under standard conditions. For heat-shock treatments (*hs-Cka* and *peb^1^* experiments), animals were maintained at 29°C throughout development.

### Cuticle preparations

Mutant embryos were processed as in [54]. Cuticles were examined under a dark field microscope.

### Molecular mapping and rescue

4.7 kb of genomic DNA encompassing the *acal* locus of homozygous embryos was PCR amplified, cloned, and sequenced. The SD08925 clone was sequenced fully. A genomic (*178D09*) and a UAS-cDNA (*UAS-acal*) rescue constructs were synthesized and employed.

### In situ hybridization and LacZ staining

For in situ hybridization, embryos were fixed, hybridized with digoxigenin-labeled RNA probes, and developed with alkaline phosphatase-NBT/BCIP. Pixel intensity was quantified using ImageJ. For LacZ staining, embryos were fixed and developed with X-Gal.

### Northern blots

Northern blots were done according to standard procedures [55]. Small RNA Northern blots were resolved by PAGE, and hybridized with labeled DNA oligonucleotides.

### Nuclear fraction preparation

Embryos were dechorionated and processed for nuclei low-salt extraction by centrifugation. Supernatant was recovered as the cytoplasmic fraction. Both fractions were homogenized in TRIzol.

### cDNA synthesis, semi-quantitative PCR, and real time PCR

RNA was purified and retro-transcribed with M-MLV reverse transcriptase. Semi-quantitative PCR and real time PCR were done by standard methods. Densitometry analysis was done using ImageJ. Primers used are listed in S2 Table.

### Fluorescence microscopy

Z-stacks of embryos undergoing DC were obtained using a Zeiss LSM 780 microscope and processed using ImageJ. Fluorescence intensity was calculated using ImageJ.

### Scanning Electron Microscopy

For imaging eyes and thoraces, animals were cold anesthetized, attached to SEM stubs with carbon paint and glue, and imaged under high vacuum in a JEOL JSM-6060.

### Thoracic cleft index and wing angle quantification

Measurements were done with iVision software.

### Adult leg preparation

Adult male thoraces were digested with potassium hydroxide and mounted for viewing and quantification of sex combs.

### Bioinformatics

*acal* homologous sequences were extracted from the Flybase Genome Browser [56]. Multiple sequence alignments were done with the CLC sequence viewer software (http://www.clcbio.com). Putative translated ORFs were analyzed with BLAST and compared in the Drosophila PeptideAtlas server [57]. Protein coding capacity was calculated according to [29].

### Statistics

All tests were done as implemented in GraphPad software or according to Kirkman, T. W. (http://www.physics.csbsju.edu/stats/).

## Supporting Information

S1 Fig Phenotypic analysis of *acal* mutants and characterization of SD08925. (A) An allelic series of embryonic cuticle defects for *∆18* (a deficiency removing a segment of *lola* and of *psq*, plus fully *acal* and *CR45135*, and expected to bear multiple defects), and *acal* mutants. Alleles are organized by decreasing embryonic lethality and frequency of phenotypes. Mutant embryos surviving embryogenesis are represented by the open spaces above the bars up to 100% in the graph, and were not studied. The *acal* allelic series deduced is: *acal^5^ >acal^6^>acal^3^ >acal^2^ >acal^4^ >acal^1^*. Embryos surviving embryogenesis are the open spaces above the bars to amount to a hundred percent. (B) Scanning electron microscope images of the *peb^1^* rough eye phenotype (smaller, disorganized ommatidia, duplicated and mis-aligned bristles) observed at the restrictive temperature (29°C), and partial suppression by heterozygosity for *acal* mutants. Scale bar is 100 μm. Anterior is left, dorsal is up. Lower panels show the positions of bristles seen in middle panels highlighting the suppression of the *peb^1^* bristle disorganization phenotype. (C) RNA from wild type embryos [E], larvae [L], pupae [P], and adults [A] analyzed by Northern blot, using a probe derived from *SD08925*, and then re-hybridized for *Rp49* without stripping. Molecular weight marker sizes are shown, and the corresponding bands for *acal* and *Rp49*. (D) *acal* semi-quantitative RT-PCR in control (*yw*) and homozygous embryos for *Δ18*, a deficiency that removes *acal*. (E) SD08925 relative expression in *acal^2^* mutants with or without the genomic rescue construct, *178D09*. n = 3 with duplicates.
(EPS)

S2 Fig *UAS-acal* expression and *acal* in situ hybridization. (A) *acal* relative expression in *acal^5^* mutant controls and in *UAS-acal* over-expressing mutant embryos, as determined by qPCR. Experiments were done three independent times, and run twice each time. Significance was determined using Student’s t-tests. (B) In situ hybridization against *acal*, in wild type *(yw)* and mutant (*acal^5^)* embryos, stage 17. Sense probes were used as negative controls. *acal^5^* homozygous embryos were selected by lack of GFP expression, present in balancer chromosomes, prior to embryo fixation and hybridization. (C) Pixel intensity quantification of the same experiment as (B). Number of animals analyzed: *yw* = 5, *acal^5^* = 6, sense = 9. Significance was assessed using Student’s t tests.
(EPS)

S3 Fig *acal* is a conserved non-coding RNA. (A) Multiple sequences alignment of the *acal* genomic locus of 12 Drosophila species. Black lines represent aligned sequences, and white spaces are gaps. On top, putative ORFs present in the *Drosophila melanogaster* locus are shown. To keep co-linearity with the genome, 5’ ends are shown to the left. Note that most ORFs present in *D. melanogaster* correspond to gaps in other species. (B) Conservation of the acal-B probe in other Drosophila species. The most conserved region corresponds in length with the band detected in small RNA Northern blots (Fig. 3E). (C) RNA was purified from wild type embryos (E), larvae (L), pupae (P), adult males (M), and adult females (F). Fragments detected with acal-A and miR-8 (control) are marked with arrowheads. Northern blots under equivalent hybridization and exposure conditions show no signal for acal-C and acal-D, except for very faint marks in the wells at the top of the lanes (arrowheads).
(EPS)

S4 Fig *acal* negatively regulates JNK signaling in the epidermis. (A–A’’, C–C’’) Wild type embryos, (B–B’’, D–D’’) *acal^5^* mutant embryos. sGMCA is a marker for cortical cytoskeleton, TRE-DsRed is a JNK activity reporter. Images are representative of five embryos per condition. Boxed areas in A and B are magnified in C and D. Note disorganized and ectopic expression of DsRed in the mutant embryo. (E) Wild type embryos, (F) *acal^2^* mutant embryos, TO-PRO-3 labels nuclei, TRE-GFP is a JNK activity reporter. Images are representative of four embryos per condition. (E’) and (F’) show the TO-PRO-3 (blue) channels of wild type and *acal^2^* embryos, respectively. (E’’) and (F’’) show the GFP (green) channels in wild type and *acal^2^* embryos, respectively. Merged images for the doubly stained embryos are shown in (E) and (F) for the same wild type and *acal^2^* embryos. Scale bars in (A, C, and E) are 50 μm. (G) Signal intensity quantification of TRE-GFP in lateral epithelia ventral to the LE, normalized against TO-PRO-3 signal intensity. Significance was assessed using Student’s t test. (H) Embryonic lethality caused by over-expression of wild type *hep (69B>hep)* is enhanced by *acal* heterozygosity. Number of animals analyzed: *69B>hep* control for *acal^1/+^; 69B>hep* = 95; *acal^1/+^; 69B>hep* = 98; *69B>hep* control for *acal^2^; 69B>hep* = 166; *acal^2/+^; 69B>hep* = 141; *acal^2/2^; 69B>hep = 93; 69B>hep* control for *acal^5/+^; 69B>hep* = 354; *acal^5/+^; 69B>hep* = 443. (I) Dorsal closure and embryonic lethality and defects seen by over-expression of a constitutively active form of *hemipterous* in the ectoderm *(69B>hep^ACT^)*, are enhanced by *acal* heterozygosity. Number of animals analysed: *69B>hep^ACT^* control for *acal^1/+^; 69B>hep^ACT^* = 263; *acal^1/+^; 69B>hep^ACT^* = 563; *69B>hep^ACT^* control for *acal^2/+^; 69B>hep^ACT^* = 321; *acal^2/+^; 69B>hep^ACT^* = 363; *69B>hep^ACT^* control for *acal^5^; 69B>hep^ACT^* = 624; *acal^5/+^; 69B>hep^ACT^* = 923; *acal^5/5^; 69B>hep^ACT^* = 153. (H-I) Significance was assessed using chi square tests, and open spaces above bars up to a hundred percent represent embryos surviving embryogenesis.
(EPS)

S5 Fig Genetic interactions between *acal* and JNK signaling components. (A) *puc^lacZ^* heterozygosity enhances the *acal* mutant phenotypes. Number of animals analyzed: *acal^1/1^ = 217, acal^1/1^;puc^lacZ/+^* = 296, *acal^2/2^* = 192, *acal^2/2^;puc^lacZ/+^* = 204, *acal^5/5^* = 391, *acal^5/5^;puc^lacZ/+^* = 178. (B) *bsk* heterozygosity significantly suppresses *acal* homozygous mutant embryos in a different genetic background (in the absence of balancer chromosomes, back-crossed to a *yw* strain). Mutant stocks were first crossed to a *yw* control strain, and then to each other. Dead embryos were analyzed. Number of animals: *bsk^1/+^ x bsk^1/+^* = 652; *bsk^1/+^*,*acal^5/+^ x bsk^1/+^* = 452; *acal^5/+^ x acal^5/+^* = 633; *acal^5/+^* *x acal^5/+^,bsk^1/+^* = 643. (C) An *acal* sensitized background does not alter the embryonic *peb^308^* mutant phenotype. Number of animals analyzed: *peb^308^/Y;/+* = 665, *peb^308^/Y; acal^5/+^* = 247.
(EPS)

S6 Fig *acal* over-expression partially rescues *raw* mutant phenotypes. (A) Control expression of *dpp* in a *yw* embryo. Expression in the leading edge is seen as a thin line surrounding the amnioserosa. (B) In an *acal^2^* mutant background, the *dpp* expression domain in the leading edge is not strongly over-expressed. (C) *dpp* in situ hybridization in *raw* mutants show robust ectopic expression of *dpp* in the lateral epithelium during dorsal closure (arrowheads; see also [19]). (D) *acal* over expression driven by the *en-gal4* driver in posterior compartments of segments rescues cell autonomously in these posterior compartments the *raw* induced *dpp* over expression (arrowheads). Number of animals analyzed: *raw^2/2^; en-gal4* = 5, *raw^2/2^; en>acal* = 5. (E) *raw^1^* heterozygosity enhances the *acal^2^* embryonic mutant phenotype. Number of animals analyzed: *acal^2/2^* = 416, *raw^1/+^,acal^2/2^* = 258, *raw^1/1^* = 278, *acal^2/+^,raw^1/1^* = 487, *acal^2/2^,raw^1/1^* = 253. (F) UAS-*acal* over-expression in the ectoderm of *raw^1^* mutant embryos, using the *69B-gal4* driver partially rescues the *raw* mutant phenotypes. Number of animals analyzed: *raw^1/1^;UAS-acal/+* = 53, *raw^1/1^; 69B-gal4/+* = 283, *raw^1/1^;69B>acal* = 28. Significance was calculated using chi square tests. (G-G’) *raw^1^* mutants show dorsal closure defects and absence of denticles. (G’) is an enlargement of (G). (H-H’) *acal* over-expression in the ectoderm of *raw^1^* mutants rescues the presence of denticles (some examples are marked by arrowheads in H-H’). (H’) is an enlargement of (H).
(EPS)

S7 Fig *lola* and *psq* over expression leads to partial larval and pupal death. (A) Cuticular analysis and quantification of *lola* and *psq* mutant embryos and larvae. Both *lola^rev6^* and *psq^rev12^* are null alleles. Number of animals analyzed: *lola^rev6/rev6^* = 366, *psq^rev12/rev12^* = 311. (B) Survival of embryos in a control stock, *yw*, where only a very small fraction (1%) die during embryogenesis. All other embryos reach the adult stage. Over expression of *lola* and *psq* via the *88A8* gene search transposon *(GS88A8)* using *69B-gal4* leads to partial larval and pupal death, but no significant changes in embryonic lethality compared to the *yw* control strain. Numbers of animals analyzed: *yw* = 526, and *69B>GS88A8* = 554. (C) qPCR of *lola* and *psq* in *acal* mutant embryos, normalized against control heterozygous siblings. Primers used detect all *lola* or all *psq* isoforms. Experiments were done three times and run in duplicate.
(EPS)

S8 Fig *acal* and *Polycomb* genetically interact. (A-B) Wing defects of *acal* or *raw* and *Polycomb (Pc)* double heterozygotes and control siblings. Arrows in (A’) and (B’) point to posterior wing compartment reduction. (C-D) *Cka* and *aop* heterozygotes do not have wing defects. (C’) *Cka* and (D’) *aop* heterozygotes also do not interact with *Pc^3^* heterozygotes in the wing. (E) *Pc^3^* control wing. (F) ‘Wing angle’ quantification in *raw, acal, Cka*, and *aop* heterozygotes with or without a mutant *Pc^3^* copy. ‘Wing angle’ was measured as in (E). (G-G’) Wild type male legs and (H-H’) extra sex combs phenotype. Numbers indicate leg identity from anterior to posterior. Asterisks mark sex combs in the first pair of male legs. (I) Quantification of the extra sex combs phenotype of *Pc^3/+^* with *raw, acal, Cka*, or *aop* heterozygosity, compared to *Pc^3/+^* siblings. Student’s t test (F, I) were used to assess significance. n = 10 for each condition.
(EPS)

S1 Table Complementation tests of *acal, lola*, and *psq* mutants. Progeny was analyzed for the presence of balancer chromosomes (CyO, i. e., heterozygotes), and flies without balancer chromosomes, i.e., heteroallelic combinations, if present. At least 100 animals were counted for each condition. When heteroallelic flies hatched, males and virgin females were crossed to *yw* adults (virgin females or males, respectively). If no progeny was observed after 4 days, heteroallelic flies were considered sterile. VF means viable and fertile, FS means female sterile, and X means non-complementing.
(DOCX)

S2 Table List of primers used in this study. Primer pairs are shown, along with the expected product size. For other applications, single primers with no product size are shown.
(DOCX)

S1 Protocol Additional materials and methods as well as additional references are provided in the S1 Protocol archive. (DOCX)
