# Correction: *acal* Is a Long Non-coding RNA in JNK Signaling in Epithelial Shape Changes during Drosophila Dorsal Closure

**DOI:** 10.1371/journal.pgen.1005138

**Published:** 2015-04-10

**Authors:** The PLOS Genetics Staff

The figure legends for Figs. 5 and 6 are incorrectly switched. The figure legend that appears with Fig. 6 should be the figure legend accompanying Fig. 5, and the figure legend that appears with Fig. 6 should be the figure legend accompanying Fig. 5. The images appear in the correct order. The authors have provided Fig. 5 and Fig. 6 with the correct figure legends here.

**Fig 5:**
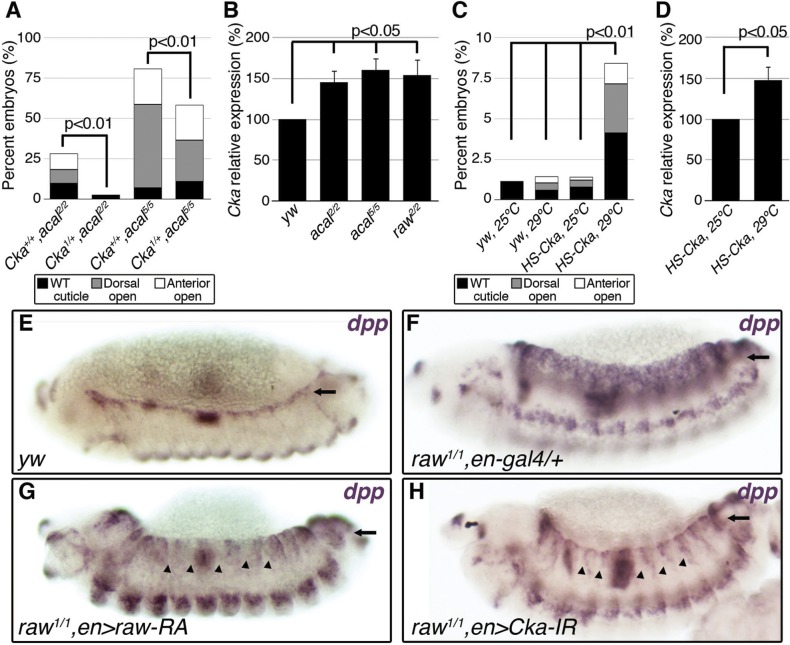
*Cka* is downstream of *raw* and *acal*. (A) Genetic interaction between *Cka* and *acal* mutants. Number of animals analyzed: *Cka* ^*+/+*^,*acal* ^*2/2*^ = 192, *Cka* ^*1/+*^,*acal* ^*2/2*^ = 219, *Cka* ^*+/+*^,*acal* ^*5/5*^ = 391, *Cka* ^*1/+*^,*acal* ^*5/5*^ = 171. (B) *Cka* relative expression in wild type and mutant embryos, as determined by qPCR. (C) Expression of a heat-shock inducible *Cka* transgene results in DC defects. Number of animals analyzed: *yw*, 25°C = 526, *yw*, 29°C = 591, *HS-Cka*, 25°C = 2006, *HS-Cka*, 29°C = 3227. (D) *Cka* expression increase due to heat shock in *hs*-Cka flies was confirmed by qPCR. For (A,C) embryos surviving embryogenesis represent the open space above the bar to amount to one hundred percent total of embryos analyzed. Chi square tests were used to calculate significance. For (B,D) represents the means of three independent experiments run twice +/− SEM. Significance was assessed using Student’s t test. (E-H) *dpp* in situ hybridization experiments, showing JNK-induced *dpp* expression (arrows). (E) Wild type embryo. (F) *raw* ^*1/1*^,*en-gal4/+* control, showing *dpp* ectopic activation (arrow). (G) Expression of *UAS-rawRA* with *en-gal 4*, which expresses *gal 4* at posterior compartments of each segment. Arrowheads show cell-autonomous suppression of *dpp* ectopic expression. (H) Silencing of *Cka* with an RNAi construct (*UAS-Cka-IR*) under *en-gal 4* also suppresses *dpp* ectopic expression in posterior compartments of *raw* ^*1*^ mutants (arrowheads).

**Fig 6:**
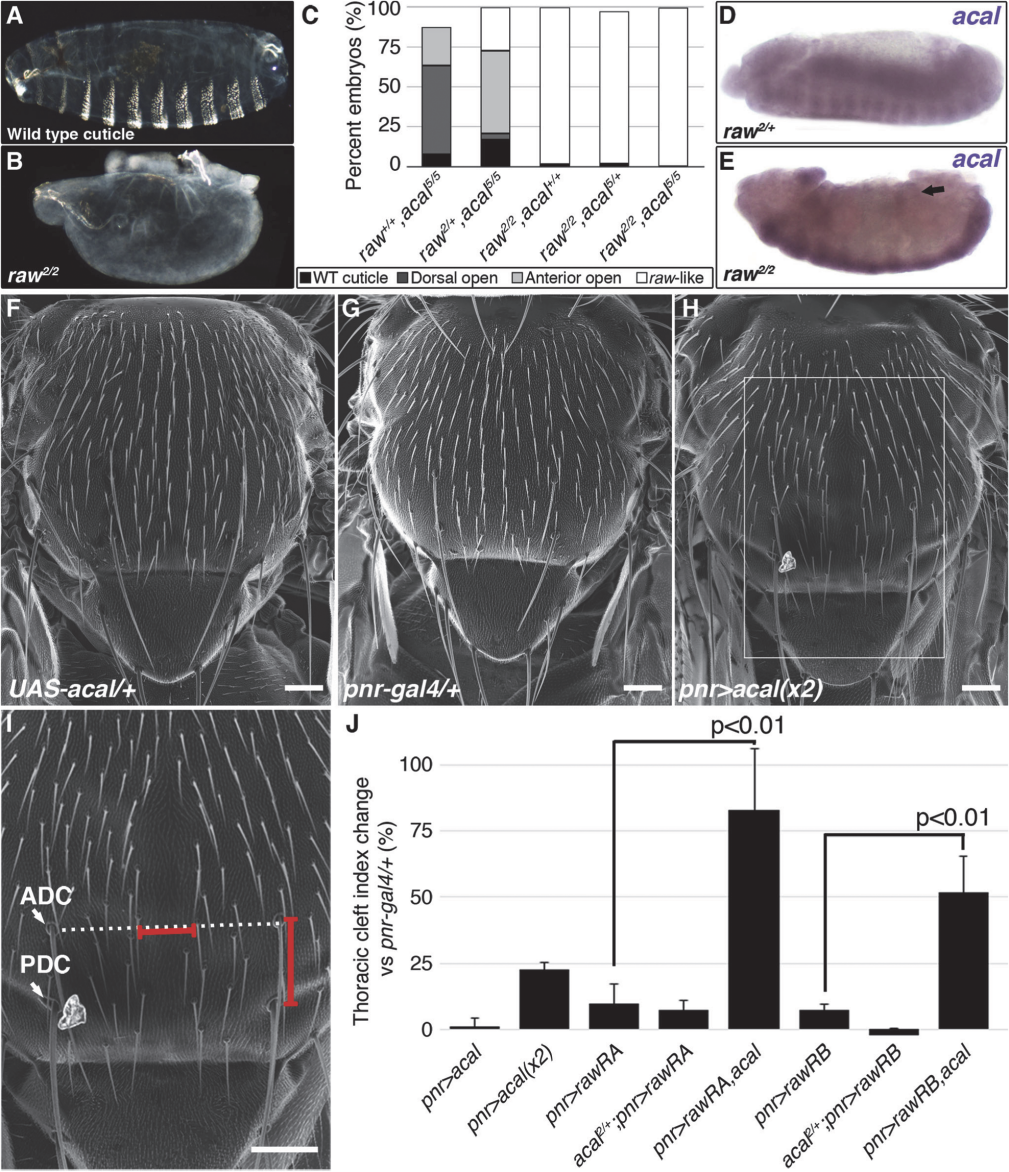
*raw* and *acal* act together to counteract JNK signaling. (A) Wild type cuticle. (B) Cuticle phenotype of *raw* mutant embryo. (C) Genetic interaction between *raw* ^*2*^ and *acal* ^*5*^ mutants. *raw*-like phenotype is depicted in (B). In *raw* ^*+/+*^; *acal* ^*5/5*^ mutants a small percentage survives embryogenesis, and constitute the open space above the bar to amount to a hundred percent total. Number of animals analyzed: *raw* ^*+/+*^,*acal* ^*5/5*^ = 391, *raw* ^*2/+*^,*acal* ^*5/5*^ = 139, *raw* ^*2/2*^,*acal* ^*+/+*^ = 366, *raw* ^*2/2*^,*acal* ^*5/+*^ = 152, *raw* ^*2/2*^,*acal* ^*5/5*^ = 208. Significance was assessed with chi square tests. (D-E) *acal* in situ hybridization in *raw* ^*2*^ mutants (n = 45; D) and heterozygous siblings (n = 106; E). Arrow in (E) points to decreased *acal* expression in the lateral epidermis. See also S5 Fig. (F-I) Scanning electron micrographs of dorsal views of adult thoraces, anterior is up. Scale bars are 100 μm. (F) *UAS-acal/+* control, (G) *pnr-gal4/+* control, and (H) over-expression of two *UAS-acal* copies. The white box in (H) is amplified in (I), depicting distances (red lines) measured to determine the thoracic cleft index, using anterior dorso-central (ADC) and posterior dorso-central (PDC) bristles as references (see Materials and Methods). (J) Percentage change of thoracic cleft index for different experimental conditions. Mean of 15 flies +/− SEM. Significance was calculated using ANOVA and Bonferroni correction.

## References

[1] Ríos-Barrera LD, Gutiérrez-Pérez I, Domínguez M, Riesgo-Escovar JR (2015) *acal* is a Long Non-coding RNA in JNK Signaling in Epithelial Shape Changes during Drosophila Dorsal Closure. PLoS Genet 11(2): e1004927 doi:10.1371/journal.pgen.1004927 25710168 10.1371/journal.pgen.1004927 PMC4339196

