# Supplementary material for: acal is a Long Non-coding RNA in JNK Signaling in Epithelial Shape Changes during Drosophila Dorsal Closure
Source: PLoS Genet. 2015 Feb 24;11(2):e1004927. doi: 10.1371/journal.pgen.1004927 (PMC4339196; doi:10.1371/journal.pgen.1004927)
Supplement: S1 Table — Progeny was analyzed for the presence of balancer chromosomes (CyO, i. e., heterozygotes), and flies without balancer chromosomes, i.e., heteroallelic combinations, if present. At least 100 animals were counted for each condition. When heteroallelic flies hatched, males and virgin females were crossed to yw adults (virgin females or males, respectively). If no progeny was observed after 4 days, heteroallelic flies were considered sterile. VF means viable and fertile, FS means female sterile, and X means non-complementing. (DOCX) [file pgen.1004927.s009.docx]

**Supporting Table S1. Complementation tests of *acal, lola,* and *psq* mutants.**

|  | *lola^00642^* | *lola^rev6^* | *acal^1^* | *acal^2^* | *acal^3^* | *acal^4^* | *acal^5^* | *acal^6^* | *psq^KG09291^* | *psq^rev7^* | *psq^rev12^* |
| --- | --- | --- | --- | --- | --- | --- | --- | --- | --- | --- | --- |
| *lola^00642^* | **X** |  | VF | VF | VF | VF | VF | VF | VF | VF | VF |
| *lola^rev6^* |  | **X** | VF | VF | VF | VF | VF | VF | VF |  |  |
| *acal^1^* |  |  | **X** | **X** | **X** | **X** | **X** | **X** | VF | VF | VF |
| *acal^2^* |  |  |  | **X** | **X** | **X** | **X** | **X** | VF | VF | VF |
| *acal^3^* |  |  |  |  | **X** | **X** | **X** | **X** | VF | VF | VF |
| *acal^4^* |  |  |  |  |  | **X** | **X** | **X** | VF | VF | VF |
| *acal^5^* |  |  |  |  |  |  | **X** | **X** | VF | VF | VF |
| *acal^6^* |  |  |  |  |  |  |  | **X** | VF | VF | VF |
| *psq^KG09291^* |  |  |  |  |  |  |  |  | **X** | VF | FS |
| *psq^rev7^* |  |  |  |  |  |  |  |  |  | **X** |  |
| *psq^rev12^* |  |  |  |  |  |  |  |  |  |  | **X** |
