# Supplementary material for: acal is a Long Non-coding RNA in JNK Signaling in Epithelial Shape Changes during Drosophila Dorsal Closure
Source: PLoS Genet. 2015 Feb 24;11(2):e1004927. doi: 10.1371/journal.pgen.1004927 (PMC4339196; doi:10.1371/journal.pgen.1004927)
Supplement: S2 Table — Primer pairs are shown, along with the expected product size. For other applications, single primers with no product size are shown. (DOCX) [file pgen.1004927.s010.docx]

**Table S2. List of primers used in this study.**

| Primer pair | Sequence | Application | Product length |
| --- | --- | --- | --- |
| acal1  acal2 | 5’-ACACGGGCAACTGAAATGATCTCACC-3’  5’-TGCCAAACGAGTTTTGGAACTCTGG-3’ | Locus sequencing | 932 |
| acal3  acal4 | 5’-GAGGGAAAGAAGAAGCAGAGG-3’  5’-GGGAACAAATTCGAGAGGCATG-3’ | Locus sequencing  Semi-quantitative PCR | 831 |
| acal5 (acalB)  acal6 | 5’-TCCCAGTGTACGAGTGATGGATGG-3’  5’-GCAGCAGGAGTTGGAAAAAGTTGGGG-3’ | Locus sequencing  In situ hybridization probe | 955 |
| acal7 (acalD)  acal8 | 5’-CCCCAACTTTTTCCAACTCCTGCTG-3’  5’-TCCCGAGCATTAGACGAAGTAGTAGC-3’ | Locus sequencing | 906 |
| acal9  acal10 | 5’-CCAACCCCTAAAACCCCGAAAACGG-3’  5’-ATGCCAGCGGGGCAACATGG-3’ | Locus sequencing | 1602 |
| banF  banR | 5’-ACCGTTCCCTTCGCACGCTT-3’  5’-CCGACTGGGATCGGTCGGCAT-3’ | Semi-quantitative PCR | 837 |
| Rp49a  Rp49b | 5’-TCAAGATGACCATCCGCCCA-3’  5’-GTTCTCTTGAGAACGCAGGC-3’ | Semi-quantitative PCR  Northern blot probe | 404 |
| Rp49b | 5’-GTTCTCTTGAGAACGCAGGC-3’ | Primer-specific cDNA synthesis | - |
| acal1 | 5’-GAGGGAAAGAAGAAGCAGAGG-3’ | Primer-specific cDNA synthesis | - |
| acalQ1  acalQ2 | 5’-CGCTGTGAAGAGTGTGAGGA-3’  5’-CATTATGATTTCGCGCCGCT-3’ | Quantitative PCR | 150 |
| ckaQ1  ckaQ2 | 5’-TTCATCCAGCACGAGTGGTC-3’  5’-CAGTGCGTACTCCAGCATCT-3’ | Quantitative PCR | 162 |
| Rp49Q1  Rp49Q2 | 5’-AGATCGTGAAGAAGCGCACC-3’  5’-ATCCGTAACCGATGTTGGGC-3’ | Quantitative PCR | 151 |
| acal-A | 5’-TTTGTGTGTGCGTGTGTGTGTGT-3’ | small RNA Northern blot probe | - |
| acal-B (acal3) | 5’-TCCCAGTGTACGAGTGATGGATGG-3’ | small RNA Northern blot probe | - |
| acal-C | 5’-CCAACATCATCATCATCATCATCAC-3’ | small RNA Northern blot probe | - |
| acal-D (acal7) | 5’-CCCCAACTTTTTCCAACTCCTGCTG-3’ | small RNA Northern blot probe | - |
| mir8 | 5’-GACATCTTTACCTGACAGTATTA-3’ | small RNA Northern blot antisense probe | - |
| lola | 5’-CTGTACAACAGGCCCATCTG-3’  5’- TGTTAATGGCATCGCTTGTG-3’ | Quantitative PCR | 59 |
| psq | 5’-ACCGGCAGCATTGAGATTAT-3’  5’-CGGGAACTGTTGCTGTAACATA-3’ | Quantitative PCR | 474 |
