# Supplementary material for: acal is a Long Non-coding RNA in JNK Signaling in Epithelial Shape Changes during Drosophila Dorsal Closure
Source: PLoS Genet. 2015 Feb 24;11(2):e1004927. doi: 10.1371/journal.pgen.1004927 (PMC4339196; doi:10.1371/journal.pgen.1004927)
Supplement: S1 Protocol — (DOCX) [file pgen.1004927.s011.docx]

**Supporting Materials and Methods.**

Genetics and fly stocks.

Besides the experiments reported in the paper, complementation crosses and embryonic phenotypical analyses were also performed in other genetic backgrounds, as a way to control for background effects and results were identical. An illustration of these is shown in Figure 4I and S5B. The mutant lines *aop^1^, bsk^1^, raw^1^,* and *raw^2^* were obtained from the Tübingen Drosophila Stock Collection. *puc^E69^* was obtained from Alfonso Martínez-Arias (University of Cambridge, UK), *peb^308^* from Howard Lipshitz (University of Toronto, Canada), *Cka^1^*, *peb^1^*, *lola^00642^, psq^KG09291^, Df(2R)BSC595, Df(2R)ED2098, Df(2R)ED2076, Pc^3^*, and *P{SUPorP}KG0911,* from the Bloomington Drosophila Stock Center (NIH P40OD018537; BDSC, #11451, #80, #10946, #14784, #25428, #9277, #8909, #1730, and #14782 respectively), *pnr^MD237^* from Ginés Morata (Universidad Autónoma de Madrid, Spain), *69B* from Andrea Brand (University of Cambridge, UK), *TRE-DsRed* and *TRE-GFP* from Dirk Bohmann (University of Rochester Medical Center, USA), *sGMCA* from Dan Kiehart (Duke University, USA), *UAS-aop^ACT^* from Ilaria Rebay (University of Chicago, USA), *UAS-rawRA* and *UAS-rawRB* from Mark Van Doren (Johns Hopkins University, USA), and *hs-Cka* from Steven Hou (NIH, USA). *Cka* RNAi from the Transgenic RNAi Project (*TRiP*, line #JF01432). *Δ18, lola^rev6^, psq^rev12^, and GS88A8* were published in [58]. Stocks were balanced with *CyO,twi>GFP,* *CyO-TM3,hs>GFP*, or *SMB6B,eve>lacZ* (BDSC #6662, #5703, and #335, respectively) to identify and discard heterozygous animals when needed. Mutant *acal* lines were backcrossed to a wild type chromosome to clean the genetic background.

Molecular mapping.

Genomic DNA from homozygous embryos was obtained by homogenizing homozygous embryos in 10 mM TRIS-HCl pH 8.2, 1 mM EDTA, 25 mM NaCl, and 0.2% Triton X-100. We then used this crude preparation to amplify PCR fragments spanning a total 4.7 kb of the *acal* locus. The oligonucleotide pairs used to amplify the locus are shown below. PCR fragments were cloned in pGEM-T Easy (Promega Corp., USA) and sequenced. Mutations were corroborated by independent DNA isolation and sequencing.

Rescue constructs.

The genomic rescue fragment was obtained from the P[acman] library project (BAC #CH322-178D09), and inserted at 76A2 by site-specific recombination with the line *PBac{y^+^-attP-9A}VK00013*. The *UAS-acal* line was generated by subcloning the EST clone SD08925 (Drosophila Genomics Resource Center, USA) in the pUAST transformation vector. The construct was injected to generate transgenic lines.

Cuticle preparations.

Embryos were collected on agar plates, and the balancer progeny was discarded. Plates were incubated for at least three days to quantify larval populations. Dead embryos were dechorionated with 50% bleach, washed in PBS and mounted in PVA and incubated at 45ºC for 24 hours to digest soft tissues. Cuticles were examined on a dark field microscope. For illustration purposes, homozygous embryos were processed in the same manner but additionally devitellinized by vigorous shaking on PBS/Methanol 1:1. Afterwards, they were mounted and photographed using a Coolsnap cf camera mounted on a Nikon Eclipse microscope (Photometrics, USA).

In situ hybridization and LacZ staining.

An *acal* 955 pb fragment was amplified by PCR (primers are described below). The product was cloned in pGEM-T Easy and used as template for probe synthesis. For *dpp*, cDNA clone RE20611 (Drosophila Genomics Resource Center, USA) was blunt-end digested with DraI, A-tailed as recommended by the pGEM-T Easy protocol, and cloned into pGEM-T Easy. Antisense and sense probes were synthesized following manufacturer’s instructions. Sense probes were negative controls. Embryos were dechorionated in 50% bleach, and fixed in 50% heptane - 7.4% formaldehyde/PBS. After, they were devitellinized in 1:1 heptane - methanol, cleared with xylene-ethanol, treated with proteinase K, and hybridized overnight with the corresponding probe. Next, embryos were washed with PBS-Tween 0.3%, incubated with preabsorbed anti-digoxigenin coupled to alkaline phosphatase antibody (Roche, Switzerland) in a 1:2000 dilution, washed, and the reaction developed with NBT/BCIP. For LacZ staining, embryos were collected and fixed with 1% glutaraldehyde and preincubated in a solution containing 10 mM NaPO_4_ pH 7.2, 150 mM NaCl, 1 mM MgCl_2_, 0.3% Triton X-100, 3.1 mM K_4_[Fe^II^(CN)_6_], and 3.1 mM K_3_[Fe^III^(CN)_6_]. The reaction was developed at 37ºC in the same solution with 0.2% X-Gal. After either X-Gal staining or in situ hybridization, embryos were mounted in Polymount and visualized in a bright field microscope.

Northern blots

We isolated RNA from *yw* embryos, larvae, pupae, and adults by homogenizing them in TRIzol. The homogenate was processed using the Direct-Zol RNA mini-prep kit. 20 µg of RNA were resolved in 0.8% agarose / 1x MOPS / formaldehyde gels and transferred to Inmobilon-NY membranes (Millipore, USA) for 40 minutes at 3 mA/cm^2^ with TBE using a semidry apparatus. RNA was fixed in a UV linker using 70,000 µJ/cm^2^ once. The membrane was then washed, and pre-hybridized with hybridization buffer (50% formamide, 5X SSC, 0.1% SDS, 0.05X Denhardt’s solution, 100 µg/mL herring sperm DNA). Probes were generated using dCTP [⍺-^32^P] and random primed using the Prime-It kit (Agilent Technologies, USA). As templates, we used 50 ng of digested SD08925, or purified *RpL30* PCR product (*Rp49,* for oligos see below). Membranes were hybridized overnight at 42 ºC, and then washed twice with 0.1% SDS, 0.1X SSC. Finally, membranes were exposed to phosphor screens for at least 7 days, and read using Typhoon 9400 or Storm 860 imaging systems. 0.5 - 10 kb RNA ladder from Invitrogen (USA) was used as reference.

Small RNA Northern blots.

We isolated RNA as above. 25 µg of RNA were resolved in 15% polyacrylamide / 8 M urea / 1x TBE gels, transferred to Immobilon-NY membranes, and fixed two times as above. Afterwards, membranes were pre-hybridized for 30 minutes with small RNA hybridization buffer (50% formamide, 5x SSPE, 5x Denhardt’s solution, 0.5% SDS, [59]). 10 pmol of oligonucleotides were labelled with ATP [γ-^32^P] using T4 polynucleotide kinase (NEB, USA). Membranes were hybridized overnight at 40ºC with the desired probe, and then washed twice in 2x SSC / 0.2% SDS, at 40ºC, 30 minutes each. Membranes were read as above. A probe against miR-8 was used as positive control, and the NEB microRNA marker or Affymetrix Low Molecular Weight markers used as references. Experiments were done 4 independent times. Oligonucleotides used as probes are described below.

Nuclear fractionation.

To obtain a nuclear-enriched RNA fraction, we modified the method described by Pazin et al. [60]. We collected 1 mg of *yw* embryos in agar plates and then dechorionated them in 50% bleach. Embryos were then homogenized in a solution containing 15 mM HEPES, 10 mM KCl, 5 mM MgCl_2_, 0.1 mM EDTA, 0.5 mM EGTA, 350 mM sucrose, 1 mM DTT, and 1 mM sodium metabisulfite. The material was filtered through sterile gauze and pelleted. The supernatant (cytoplasmic fraction) and the pellet (nuclear fraction) were homogenized independently in TRIzol (Invitrogen), and RNA purified using the Direct-Zol RNA mini-prep kit.

Retro-transcription , quantitative, and semi-quantitiatve PCR.

RNA purification was done using TRIzol and Direct-zol RNA mini-prep kit with in-column DNase digestion. We used 2 μg of RNA to synthesize cDNA with M-MLV reverse transcriptase. Mock reactions without enzyme were performed in parallel to control for genomic DNA contamination. For quantitative PCR experiments, we used Maxima SYBR Green qPCR master mix (Fermentas, USA) or FastStart DNA Master SYBR Green I (Roche, Switzerland) in a LightCycler 1.0 instrument (Roche, Switzerland). We generated oligo-dT cDNA for *Cka* and *aop* quantification, and primer-specific cDNA for *acal*. In the former case, we also generated primer-specific cDNA for *Rp49* using the same RNA sample, to allow direct comparisons between the two primer-specific cDNAs. Oligonucleotides used are listed below.

Anti-GFP and TO-PRO-3 staining

Embryos were dechorionated, fixed in formaldehyde, and de-vitellinized according to [61], except that embryos were treated in methanol only once, and then rehydrated in phosphate buffered saline with 0.3% Triton-X100 afterwards. Primary antibody used was rabbit anti-GFP (1:100, #SC-8334, Santa Cruz Biotechnology, pre-absorbed with fixed wild type embryos in incubation solution at least one hour before use), and anti-rabbit coupled to Alexa 488 (Molecular Probes), 1:1000 (similarly pre-absorbed with fixed wild type embryos). The last wash had 2.5 µM TO-PRO-3 (Molecular Probes) to stain nuclei. Embryos were imaged using a Zeiss 780 confocal microscope.

Thoracic cleft index calculation.

Female flies were euthanized with ethyl acetate and then immobilized with entomology pins. Thoraces were photographed in a Zeiss Lumar.V12 stereoscope with 63x magnification, equipped with a Coolsnap cf camera. We employed the iVision software to measure the distance without microchaetae between the most central microchaete rows in the dorsal thorax, at the level of the anterior dorsocentral macrochaetes. This value was normalized to the distance between the anterior and posterior dorsocentral macrochaetes, to adjust for animal size. We termed this value “thoracic cleft index”. For illustrative purposes, animals were subjected to SEM as described above.

Wing angle calculation.

Female flies were photographed as above, focusing on the wings. We employed the iVision software to measure the angle formed between the L1-wing margin intersection to the L2-wing margin intersection, and form the L2-wing margin intersection to the L3-wing margin intersection. We used reference points to mark the angle in the wings to control for wing size differences, irrespective of whether they are located on the anterior or posterior territories of the wing, as alterations in one compartment would reflect on the other.

Ectopic sex combs quantification.

Male flies were fixed in ethanol-glycerol, and the legs attached to the thoracic segment were then cleared in 10% KOH for 10 minutes at 100ºC. Legs were then dehydrated in ethanol and mounted in ethanol-lactic acid. Sex combs were analyzed in a Nikon Eclipse light microscope.

Bioinformatics.

Multiple sequence alignments were done with CLC, with standard gap settings and the slow alignment option. The similarity tree was created with CLC, performing bootstrap analysis of 10000 replicates, and default settings. CLC was used to find ORFs with a minimum length of 30 nucleotides. Presence of Kozak sequences was assayed using the Salamov et al. program [62]. Conservation plots were generated with CLC or taken from the UCSC genome browser [<http://genome.ucsc.edu>, [63]. Putative translated ORFs were subjected to BLAST using the UniProtKB database [64]. Some queries showed hits with p>5 and were considered not significant. For coding potential calculation, reference sequences were taken from Flybase, using the longest transcripts available, and for *acal* the SD08925 sequence.

Statistics.

Chi-square tests were used to determine the significance of differences between expected and observed proportions of embryonic phenotypes. Thoracic cleft index change was compared using ANOVA with the Bonferroni correction to adjust for multiple comparisons. qPCR experiments were compared using Student’s t-test. The Kolmogorov-Smirnov test was used to compare the distribution of TRE-Ds.Red fluorescence intensity in mutant and control embryos.

**Supporting References**

58. Ferres-Marco D, Gutierrez-Garcia I, Vallejo D, Bolivar J, Gutierrez-Aviño F, et al. (2006) Epigenetic silencers and Notch collaborate to promote malignant tumours by Rb silencing. Nature 439: 430-436.

59. Pall GS, Hamilton AJ (2008) Improved northern blot method for enhanced detection of small RNA. Nat Protoc 3: 1077-1084.

60. Sullivan W, Ashburner M, Hawley RS (2000) Drosophila protocols. Cold Spring Harbor, N.Y.: Cold Spring Harbor Laboratory Press. xiv, 697 p. p.

61. Karr TL, Alberts BM (1986) Organization of the cytoskeleton in early Drosophila embryos. J Cell Biol 102: 1494-1509.

62. Salamov AA, Nishikawa T, Swindells MB (1998) Assessing protein coding region integrity in cDNA sequencing projects. Bioinformatics 14: 384-390.

63. Karolchik D, Barber GP, Casper J, Clawson H, Cline MS, et al. (2014) The UCSC Genome Browser database: 2014 update. Nucleic Acids Res 42: D764-770.

64. UniProt C (2013) Update on activities at the Universal Protein Resource (UniProt) in 2013. Nucleic Acids Res 41: D43-47.
